# Absorption, distribution, metabolism, and excretion of [^14^C]SH-1028, a third-generation *EGFR*-TKI, in rats and humans

**DOI:** 10.3389/fphar.2026.1897957

**Published:** 2026-08-12

**Authors:** Sufeng Zhou, Yuping Wu, Wei Liu, Chen Zhou, Lijun Xie, Quankun Zhuang, Lu Wang, Juan Chen, Bei Zhu, Chen Zhang, Xiaomeng Zhang, Feng Shao

**Affiliations:** 1 Phase I Clinical Trial Unit, The First Affiliated Hospital with Nanjing Medical University, Nanjing, China; 2 Department of Clinical Pharmacology, Pharmacy College, Nanjing Medical University, Nanjing, China; 3 Nuclear Medicine Department, The First Affiliated Hospital with Nanjing Medical University, Nanjing, China; 4 Nanjing Sanhome Pharmaceutical Co., Ltd., Nanjing, China

**Keywords:** mass balance, metabolism, NSCLC, QWBA, SH-1028

## Abstract

**Introduction:**

SH-1028 is an irreversible third-generation epidermal growth factor receptor-tyrosine kinase inhibitor (EGFR-TKI) developed for treating locally advanced or metastatic non-small cell lung cancer (NSCLC) harboring *EGFR* T790M mutation. This study aimed to systematically investigate the absorption, distribution, metabolism and excretion (ADME) profiles of SH-1028 in rats and humans using a [^14^C] radiolabeled tracer.

**Methods:**

This integrated preclinical and clinical investigation comprised a radiolabeled mass balance study with quantitative whole-body autoradiography (QWBA) in rats, followed by a human mass balance study of [^14^C]SH-1028. Blood, urine and fecal samples were collected at predefined time points or intervals. Plasma pharmacokinetics, mass balance, metabolite profiling, and structural identification of major metabolites were assessed. Safety was also evaluated throughout the clinical phase.

**Results:**

Following a single oral dose of [^14^C]SH-1028 in humans, the mean total recovery of radioactivity was 85.02%, with 81.55% in feces and 3.48% in urine, indicating fecal excretion as the primary elimination pathway, which is consistent with observations in rats. Two major circulating metabolites not detected in animal studies, M541i and Imp2, were identified in human plasma, accounting for 20% and 60% of the total plasma radioactivity, respectively. The cumulative excretion of unchanged SH-1028 in urine and feces accounted for only 3.52% of the administered dose, indicating that SH-1028 undergoes extensive metabolism *in vivo* and is eliminated primarily as metabolites.

**Conclusion:**

SH-1028 was well tolerated in subjects after a single oral administration of 200 mg containing 3.26 MBq (88 μCi) of [^14^C]SH-1028. The parent drug and its metabolites M541i and Imp2 were identified as the major circulating components. Mass balance studies in both rats and humans consistently demonstrated that SH-1028 is eliminated predominantly via fecal excretion, undergoes extensive metabolism, and is cleared primarily as metabolites.

## 1 Introduction

Lung cancer is one of the malignant tumors with the highest morbidity and mortality rates worldwide, posing a severe threat to human health. Among its subtypes, non-small cell lung cancer (NSCLC) accounts for about 85% of all cases (Molina et al., 2008; Zappa and Mousa, 2016). In NSCLC patients, epidermal growth factor receptor (*EGFR*) mutations represent the most common driver gene alteration in East Asian populations, with a reported frequency of 22.2%–64.2%, significantly higher than in Caucasians (approximately 10%) (Ke and Wu, 2016). In recent years, targeted drugs, primarily signal transduction inhibitors, have demonstrated remarkable clinical efficacy by specifically blocking tumor-proliferation pathways (Herbst et al., 2018; Imyanitov et al., 2021). Notable progress has been made with inhibitors directed against oncogenic drivers such as EGFR, anaplastic lymphoma kinase (ALK), and c-ros oncogene 1 (ROS1), as well as immunotherapies, all of which have significantly improved outcomes for patients with advanced NSCLC (Li et al., 2021; Drilon et al., 2020; Howlader et al., 2020).

EGFR-tyrosine kinase inhibitors (TKIs) have become the standard first-line treatment for NSCLC patients with *EGFR*-sensitizing mutations. First-generation EGFR-TKIs, such as gefitinib, erlotinib and icotinib, have significantly improved outcomes for these patients (Ke and Wu, 2016; Goss et al., 2016; Lee, 2017). However, most patients eventually develop acquired resistance to first-generation TKIs. The T790M mutation in exon 20 of the *EGFR* gene is the primary mechanism for this acquired resistance, which is detected in approximately 50%–60% of such patients (Lee et al., 2017). Second-generation EGFR-TKIs (e.g., afatinib) provide limited clinical benefit against T790M. Therefore, third-generation EGFR-TKIs (such as osimertinib, almonertinib, furmonertinib, lazertinib) have been successfully developed. These agents can specifically and potently inhibit both the T790M mutation and primary sensitizing mutations, and are now the preferred standard treatment for T790M-positive patients (Ward et al., 2013; Bao et al., 2016; Shi et al., 2020; Lee et al., 2022; Soria et al., 2018). Furthermore, they have also shown superior efficacy against brain metastases (Zalaquett et al., 2023; Lu et al., 2022).

SH-1028 (Rilertinib) is a novel third-generation EGFR-TKI developed by Nanjing Sanhome Pharmaceutical Co., Ltd. It was approved by the China National Medical Products Administration (NMPA) in June 2024 for the treatment of locally advanced or metastatic NSCLC harboring *EGFR* T790M mutation. As an irreversible inhibitor based on a pyrimidine skeleton, SH-1028 retains the typical characteristics of this class. The structure is derived from osimertinib though the replacement of methyl indole ring with a 6,7,8,9-tetrahydropyrrole [1,2-a] indole group, while retaining the core pyrimidine ring (Han et al., 2021). This modification was designed to enhance metabolic stability and potentially reduce wild-type EGFR inhibition through unique metabolic pathways. Preclinical and clinical studies have demonstrated that SH-1028 exhibits significant antitumor activity and a manageable safety profile (Han et al., 2021; He et al., 2023).

The pharmacokinetic (PK) profiles of SH-1028 have been preliminarily characterized in both preclinical and early-phase clinical studies. *In vitro* studies have demonstrated that SH-1028 is primarily eliminated through phase I metabolic pathways, including demethylation, oxidation, and dealkylation (Li et al., 2023). A total of 27 metabolites were identified, with no human-specific metabolites detected across the tested species (Han et al., 2021). Among these, the demethylated metabolite M525c (Imp3, active metabolite) was identified as a major metabolite (Han et al., 2021). Further *in vitro* studies confirmed that SH-1028 is mainly metabolized by CYP3A4. It exhibits mild inhibitory effect on CYP1A2, 2B6, 2C19, 2D6 and 3A4, and has a potential inductive effect on CYP3A4 mRNA expression (Li et al., 2023). However, the risk of clinically significant drug-drug interactions (DDIs) at therapeutic exposures is considered low (Li et al., 2023). A phase I dose-escalation study in patients with EGFR T790M-positive advanced NSCLC (NCT03603262) revealed linear PK characteristics of SH-1028. Following a single dose, the time to reach peak plasma concentration (T_max_) ranged from 3.0 to 6.0 h, and the mean terminal elimination half-life (t_1/2_) was between 15.0 and 19.09 h (He et al., 2023). Notably, the systemic exposure of the main metabolite Imp3 was only about 55% relative to the parent drug. In contrast, the exposure to another metabolite, M468 (Imp2, an inactive metabolite), was approximately 4.4-fold that of the parent drug, suggesting that Imp2 may be the predominant circulating metabolite in humans (Zhou et al., 2023). This finding was corroborated in the phase I DDI study (CTR20210558), which also reported a high exposure level of Imp2 in humans (Li et al., 2023). In summary, while preliminary data are available, the complete metabolic profile, primary elimination pathways, and mass balance characteristics of SH-1028 in humans have not been fully elucidated. Therefore, a definitive human mass balance study is required to clarify these PK questions.

The human radiolabeled mass balance study is considered to be the gold standard for directly and comprehensively obtaining the absorption, distribution, metabolism and excretion (ADME) profiles of a drug in humans. The data generated from such a study are not only important for drug safety evaluation but also provide key evidence for dosing adjustment in patients with renal or hepatic impairment and for the assessment of DDIs risks. Comparing these data with preclinical results will establish a systematic and complete PK rationale to support the appropriate clinical use of SH-1028.

Based on the above considerations, to systematically elucidate the *in vivo* disposition of SH-1028, this study integrated preclinical and clinical investigations. First, a radiolabeled mass balance study coupled with quantitative whole-body autoradiography (QWBA) was performed in rats to obtain its complete preclinical disposition profile. Then, to directly elucidate the PK, mass balance, and metabolic pathways of SH-1028 in humans, a phase I mass balance study (NCT04810533) was conducted in healthy male subjects following a single oral dose of [^14^C]SH-1028.

## 2 Materials and methods

### 2.1 Chemicals, materials and drug formulation

The [^14^C]SH-1028 used in both the clinical study (specific activity: 2109.74 MBq/mmol; radiochemical purity: 98.74%) and the rat QWBA study (specific activity, 2162.65 MBq/mmol; radiochemical purity, 98.53%) was provided by Wuxi Beta Pharmaceutical Technology Co., Ltd. And the [^14^C]SH-1028 used in the rat pharmacokinetic, excretion, and metabolism studies (radiochemical purity >99%) was obtained from WuXi AppTec Co., Ltd. A site-specific ^14^C-labeling strategy was employed in the synthesis of [^14^C]SH-1028.

**FIGURE 1:**
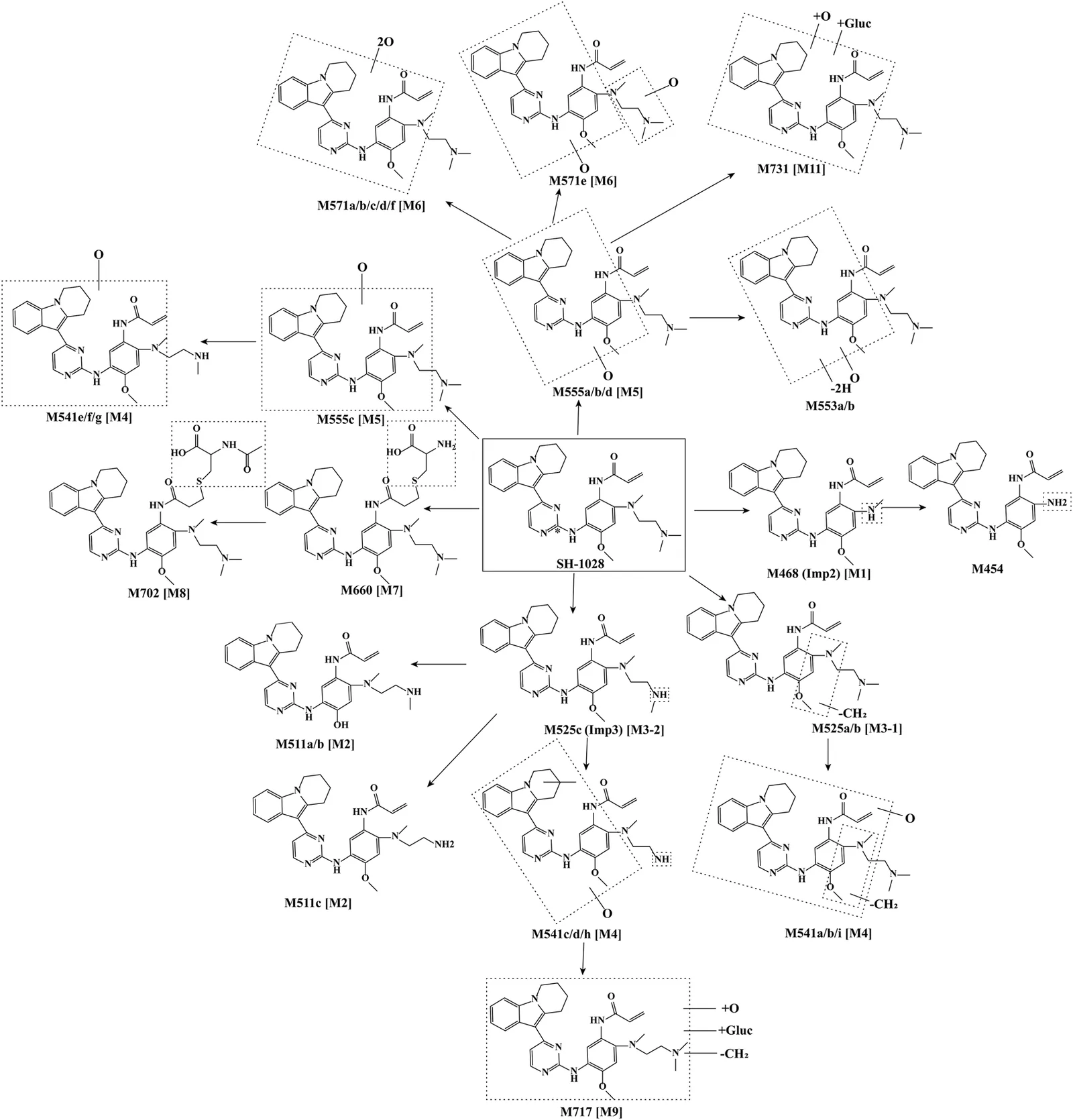
Primary metabolic pathway of [^14^C]SH-1028 in humans and rats (square brackets indicate rat metabolites). *Position of ^14^C-label.

The oral formulation of [^14^C]SH-1028 for clinical administration was prepared by Nanjing Meixinnuo Pharmaceutical Technology Co., Ltd. Unlabeled SH-1028 (chemical structure and labeling position of ^14^C are depicted in Figure 1) and its related metabolites were synthesized and supplied by Nanjing Sanhome Pharmaceutical Co., Ltd. All other chemicals and solvents were of analytical grade or higher and purchased from commercial suppliers.

The investigational product was manufactured under Good Manufacturing Practice (GMP)-compliant conditions. The clinical dosing formulation was prepared using an isotope dilution approach, in which [^14^C]SH-1028 and unlabeled SH-1028 were separately dissolved in ethanol and a small amount of hydrochloric acid. Based on the predefined clinical dose regimen, appropriate amounts of labeled and unlabeled SH-1028 solutions were combined to obtain a homogeneous [^14^C]SH-1028 solution. This solution was then uniformly blended with pharmaceutical excipients, and the resulting mixture was processed under reduced pressure via rotary evaporation to yield the final oral dosage form. The investigational product was prepared as a single 200 mg oral dose containing 3.26 MBq of [^14^C]SH-1028 and stored at −10 to −30 °C. Before administration, the formulation was reconstituted with 40 mL of drinking water at the clinical site. The dosing container was rinsed three times with an additional 200 mL of water, and all rinses were administered to ensure complete dose delivery. After administration, dosing containers were retained, and residual radioactivity was quantified to verify the actual administered dose, which served as the basis for calculating the percentage of dose recovered in excreta.

### 2.2 Ethics approval

All animal experiments were conducted in accordance with the guidelines of the Association for Assessment and Accreditation of Laboratory Animal Care International (AAALAC). The experimental protocols were reviewed and approved by the Institutional Animal Care and Use Committee.

The clinical study (ClinicalTrials.gov Identifier: NCT04810533) was conducted at the Phase I Clinical Trial Unit of the First Affiliated Hospital with Nanjing Medical University. It was conducted in accordance with the Declaration of Helsinki, Good Clinical Practice (GCP) ethical guidelines and the relevant regulations. The study protocol, amendments, informed consent forms and other study-related documents were reviewed and approved by the Ethics Committee of the hospital. Written informed consent was obtained from all participants before the initiation of any study-related procedures.

### 2.3 Rat ADME study

#### 2.3.1 Animal sources

Sprague-Dawley (SD) rats used for rat PK, excretion and metabolism studies were purchased from Shanghai SLAC Laboratory Animal Co., Ltd. (Certificate No. 2015000528177). Long-Evans (LE) and SD rats used for the QWBA study were purchased from Army Medical University (Certificate No. 0008099) and Beijing Vital River Laboratory Animal Technology Co., Ltd. (Certificate No. 33000800000719), respectively. For the QWBA study, both rat strains were used to comprehensively evaluate tissue distribution, with particular emphasis on melanin-associated retention in pigmented tissues, and with SD rats serving as the standard reference strain.

#### 2.3.2 Study design

The preclinical phase comprised a PK study, a mass balance study, and a QWBA study, all conducted in rats.

##### 2.3.2.1 PK and mass balance study

The PK study was conducted in six SD rats (three males and three females). Each animal received a single oral dose of 7.18 MBq (194 μCi)/15.0 mg/kg [^14^C]SH-1028. Serial blood samples were collected at scheduled time points for up to 48 h post-dose. The mass balance study was conducted in twelve SD rats (six males and six females). Each animal received a single oral dose of 6.96 MBq (188 μCi)/14.6 mg/kg [^14^C]SH-1028. Half of the animals (N = 6) underwent bile duct cannulation. Urine and feces were collected from non-cannulated rats at designated intervals for up to 168 h post-dose. For bile duct-cannulated rats, bile samples were collected for up to 48 h. Rat plasma concentrations of the unlabeled SH-1028 were determined using a validated liquid chromatography-tandem mass spectrometry (LC-MS/MS) method. Total radioactivity in plasma, urine, feces and bile samples were measured by liquid scintillation counter (LSC). Metabolite profiling and identification were performed on pooled plasma, urine, feces and bile samples.

##### 2.3.2.2 QWBA study

It was conducted in seven male Long-Evans (LE) rats and ten SD rats (five males and five females). Each animal received a single oral dose of [^14^C]SH-1028 at 3.7 MBq (100 μCi)/15 mg/kg. Rats were anesthetized with carbon dioxide at 0.5, 6, 24, 72, 168 (LE only), and 504 h (LE only) post-dose, and whole blood samples were collected via cardiac puncture. The carcasses were then immediately frozen in a dry ice bath containing n-hexane. For QWBA analysis, radioactivity concentrations in selected tissues and regions were quantified by region-of-interest analysis using AIDA software (version 5.1; Raytest Isotopenmessgeräte GmbH, Straubenhardt, Germany).

Detailed procedures for sample collection and preparation, chromatographic separation, spectral and QWBA analyses are provided in the Supplementary Material.

### 2.4 Human mass balance study

#### 2.4.1 Study design

This phase I, single-center, open-label, single-dose clinical study was conducted to evaluate the mass balance, metabolic transformation and excretion pathways of [^14^C]SH-1028 following a single oral dose in healthy Chinese male subjects (Supplementary Figure S1). This study was conducted from November 2020 to March 2021.

A total of four healthy male subjects were enrolled and received a single oral dose of 200 mg containing 3.26 MBq (88 μCi) of [^14^C]SH-1028 as an oral suspension. The enrolled subjects were admitted to the Phase I Clinical Unit 2 days prior to doing (D-2) and fasted overnight for at least 10 h before drug administration, with water restricted 1 h before and after dosing. On the day of dosing, subjects were admitted to the nuclear medical ward and received the [^14^C]SH-1028 suspension at 8:00 a.m. (±30 min). After 2 days of the administration, subjects were transferred back to Phase I Clinical Unit, and were resident in the unit for up to 15 days post-dose. The length of the residential period could be reduced or extended depending on the radioactivity levels in blood (plasma) and excreta. Physical examinations, vital sign monitoring, 12-lead ECG, collection of blood and excreta samples, and collection of samples for clinical laboratory were performed at scheduled time points before and after drug administration. Blood sample collection was terminated when the radioactivity concentration in two consecutive time points fell below twice the background level of the blood sample. Urine and fecal sample collection was terminated when the cumulative radioactivity in excreta exceeded 80% of the administered dose, and the excretion in two consecutive days was less than 1% of the administered dose. All subjects were monitored for adverse events (AEs) and the use of concomitant medications throughout the study.

#### 2.4.2 Study population

Healthy adult male subjects aged 18–50 years, with a body weight ≥50 kg and a body mass index (BMI) ranging from 19.0 to 26.0 kg/m^2^, were eligible for enrollment. Eligible individuals were required to be in good health as confirmed by a comprehensive medical evaluation, including physical examination, vital signs, electrocardiogram, and clinical laboratory tests (hematology, urinalysis, hepatic function). The key exclusion criteria included abnormalities in the neurological, respiratory, cardiovascular, digestive, hematologic, lymphatic, endocrine, or musculoskeletal systems that were deemed clinically significant by the investigator. Detailed inclusion and exclusion criteria are shown in the Supplementary Material.

#### 2.4.3 Sample collection

Blood samples were collected at 0.5 h before dosing and at 1, 2, 3, 4, 6, 8, 12, 24, 48, 72, 120, 168, 216, 264 and 336 h after dosing. At each time point, a portion of the blood sample was immediately aliquoted into a cryovial for measuring total radioactivity in whole blood. The remaining blood was transferred into K_2_-EDTA anticoagulant collection tubes. After centrifugation, the supernatant was collected for analysis of unlabeled compound, total radioactivity and metabolite profiling in plasma.

Urine was collected on the day before dosing, and during the intervals of 0–4 h, 4–12 h, and 12–24 h after dosing, and then every 24 h thereafter up to the end point of excreta. Urine samples collected during specified time intervals were pooled into a 5 L plastic container. Appropriate anti-adsorbent agents could be added as needed for sample analysis. And the pooled urine was used for the determination of total radioactivity, metabolite profiling and identification.

Individual Feces were collected at predose (24 h before dosing) and then every 24 h thereafter up to the end point of excreta. Fecal homogenates were subjected to total radioactivity measurement, metabolite profiling and identification.

All samples were stored at −20 °C and transferred to Nanjing Meixinnuo Pharmaceutical Technology Co., Ltd. for bioanalysis.

#### 2.4.4 Sample analysis

##### 2.4.4.1 Determination of unlabeled SH-1028 and its metabolites in plasma

Plasma concentrations of unlabeled SH-1028 and its metabolites (Imp2 and Imp3) were determined using a validated LC-MS/MS method. The LC-MS/MS system was conducted with a Sciex API 5000 tandem MS (Applied Biosystems, CA, USA) coupled with a 30-AD XR HPLC system (Shimadzu Co., Kyoto, Japan). A 50 μL aliquot of the plasma sample was transferred to a 96-well plate. Then, 200 μL of acetonitrile and 50 μL of internal standard were added and the mixture was vortexed for protein precipitation. After centrifugation at 4 °C and 3200 g for 10 min, a 100 µL aliquot of the supernatant was collected. Ammonium formate solution was added, and the mixture was vortexed prior to injection into the LC-MS/MS system.

Chromatographic separation was achieved on an ACE 3 EXCEL C18-AR column (5 μm, 50 × 2.1 mm) maintained at 40 °C. The mobile phase consisted of A (water containing 0.2% formic acid) and B (acetonitrile), delivered a flow rate of 0.5 mL/min. A gradient elution program was used as follows: 0–0.8 min, 28%B; 0.8–2.0 min, 28%B→95%B; 2.0–2.9 min, 95%B; 2.9–3.0 min, 95%B→28%B; 3.0–4.5 min, 28%B. The ionization was conducted in positive ion mode using the selected transitions m/z 540.5→72.2 for SH-1028, m/z 469.4→383.4 for Imp2 and m/z 526.4→469.4 for Imp3. The calibration range was from 0.200 ng/mL to 200 ng/mL for SH-1028 and Imp3, and from 0.400 ng/mL to 400 ng/mL for Imp2. The lower limit of quantitation (LLOQ) was 0.200 ng/mL for SH-1028 and Imp3, and 0.400 ng/mL for Imp2.

##### 2.4.4.2 Measurement of total radioactivity

Total radioactivity in plasma, whole blood, urine and feces were determined by Tri-Carb model 3110 TR LSC (Perkin Elmer, Wellesley, MA) with automatic external standard quench correction.

For plasma and urine samples, aliquots (approximately 300 µL of plasma or 5 mL of urine, in duplicate for urine) were transferred to scintillation vials, accurately weighed, mixed with scintillation cocktail, and analyzed by LSC following vortex mixing. For whole blood and fecal samples, combustion was required prior to LSC analysis. Approximately 300 µL aliquots of whole blood were transferred to combustion boats, weighed and dried in a fume hood. The dried residues were taken for combustion with oxygen. For feces, homogenization was performed by soaking in 50% aqueous isopropanol with the total weight recorded. Duplicate aliquots of approximately 0.3 g were then weighed into combustion boats, air-dried, combusted with oxygen and finally analyzed by LSC.

##### 2.4.4.3 Metabolite profiling and identification

Two pooled approaches were applied for the analysis of plasma metabolite profiling. The first method was equal-volume pooling. Plasma samples collected at 2 h, 4 h, 8 h, 12 h and 48 h post-dose from four subjects were separately pooled by time point, resulting in five pooled samples. The second method was according to the AUC pooling formula (Hop et al., 1998), plasma samples from pre-dose and 1, 2, 3, 4, 6, 8, 12, 24, 48, 72, 120 and 168 h post-dose were pooled to obtain one representative pooled sample to analyze. An aliquot of the pooled plasma was mixed with 3-fold volume (v/v) of methanol: acetonitrile (7:3, v/v), vortexed for 1 min, shaken for 10 min, and centrifuged at 4 °C and 4,000 rpm for 10 min. The residue was re-suspended with no more than 1-fold volume of water, then extracted twice with 2-fold volume of methanol-acetonitrile (7:3, v/v). The combined supernatants were evaporated for dryness under nitrogen. And the residue was redissolved in methanol-acetonitrile-water (5:2:3, v/v/v) for metabolite profiling.

Urine samples from each subject were first pooled into three intervals: 0–12 h (0–4 and 4–12 h), 12–48 h (12–24 and 24–48 h), and 48–144 h (48–72, 72–96, 96–120, and 120–144 h). For each interval, equal percentage volumes from all four subjects were then combined across subjects. The resulting interval pools were finally mixed equally to obtain a total pooled urine sample. The pooled sample was enriched and purified by solid-phase extraction (SPE) cartridge (Oasis HLB, 6 cc, 200 mg). The cartridge was conditioned sequentially with methanol and water. After loading 30 mL of urine and washing with pure water, the loading and washing fractions were collected. The analytes were eluted with methanol, and radioactivity was measured in each fraction to calculate extraction recovery. The methanol eluate was dried under nitrogen and reconstituted in methanol-acetonitrile-water (4:2:4, v/v/v) for metabolite profiling.

Fecal samples were pooled using an equal-weight percentage method. For each subject, feces collected during the 0–24 h, 24–48 h, and 48–120 h (including 48–72, 72–96, and 96–120 h) intervals were separated as three individual interval fractions. Equal percentages of the total weight from all four subjects were combined within each interval, generating three cross-subject pooled samples. These interval pools were then mixed in equal weight percentages to obtain a total pooled sample. An appropriate amount of pooled feces samples was mixed with 3-fold volume of methanol-acetonitrile (3:7, v/v). The mixture was vortexed for 2 min and centrifuged at 4 °C and 4,000 rpm for 15 min. Then the residue was processed following the same procedure as described for the plasma samples.

Metabolite profiling was performed using a Shimadzu UFLC-20A system. Chromatographic separation was achieved on a column of ACE C18-AR column (150 × 4.6 mm) maintained at 35 °C. The mobile phase consisted of A (water containing 0.2% formic acid and 5 mM ammonium formate) and B (acetonitrile/water (95:5, v/v) containing 0.1% formic acid and 2 mM ammonium formate) with a flow rate of 0.7 mL/min. The gradient started as 13% B from 0.01 to 3 min, 13%–30% B from 3 to 30 min, 30%–55% B from 30 to 48 min, 55%-90%B from 48 to 53 min, 90% B from 53 to 58 min, 90%–13% B from 58 to 58.1 min. HPLC effluent was collected into 96-well LunaPlate™ at a rate of 15s/well for metabolite profiling. The 96-well plate was dried using a SpeedVac system, and the radioactivity in each well was analyzed by solid scintillation counting. The resulting data were processed with ARC® Convert and Evaluation software to generate radiochromatograms for metabolite profiling.

Meanwhile, selected samples were analyzed by HPLC coupled with the radioactivity detector or high-resolution mass spectrometry (LC-RAM/HRMS). Metabolite identification was performed based on chromatographic retention behavior, mass spectrometric data, and comparison with available reference standards as well as structural information of metabolites obtained from previous studies (Li et al., 2023).

#### 2.4.5 PK analysis

PK parameters for unlabeled SH-1028 and its metabolites (Imp2 and Imp3) in plasma, and for total radioactivity in blood and plasma were calculated by a non-compartmental approach using WinNonlin software v7.0 (Pharsight Corporation, Mountain View, CA). C_max_ (peak plasma concentration after administration) and T_max_ (time to reach C_max_) were obtained directly from the observed concentration-time data. t_1/2_ (terminal elimination half-life) was calculated as ln2/λ_z_, where λ_z_ was the terminal elimination rate constant. The AUC_0-t_ (area under the concentration-time curve from zero to the last measurable time point) was estimated using the linear trapezoidal rule and ${\text{AUC}}_{0-\infty }$ (AUC from zero to infinity) was calculated as AUC_0-t_ + C_t_/λ_z_, where C_t_ was the last detected concentration. CL/F (apparent oral clearance) and V_d_/F (apparent volume of distribution) were estimated as Dose/ ${\text{AUC}}_{0–\infty }$ and CL/λ_z_, respectively.

#### 2.4.6 Safety assessment

Safety for the human mass balance study was monitored and evaluated based on vital signs, 12-lead ECGs, as well as the study of blood chemistry, urinalysis and hematology. All AEs encountered during the study were monitored and recorded in detail. Each AE was evaluated by the investigator with regard to its intensity, duration, outcome or the potential relationship to the study drug.

## 3 Results

### 3.1 Rat ADME results

#### 3.1.1 PK results

Following a single oral dose of 7.18 MBq (194 μCi)/15 mg/kg [^14^C]SH-1028 to rats, the plasma concentration-time profiles for unlabeled SH-1028 and total radioactivity are presented in Supplementary Figure S2, and the corresponding PK parameters are summarized in Table 1. The total radioactivity in plasma peaked at 4 h post-dose, with a C_max_ of 694 ng eq./mL and a AUC_0–48 h_ of 11917 h ng eq./mL. The unlabeled SH-1028 reached its maximum concentration at 5 h, with a C_max_ of 236 ng/mL, and the mean AUC_0-t_ was 1837 h ng/mL accounting for only 15.4% of the total radioactivity. In addition, the t_1/2_ of the total radioactivity and unlabeled SH-1028 were 18.7 h and 2.80 h, respectively, indicating the presence of metabolites that are eliminated more slowly than SH-1028 in the systemic circulation.

**TABLE 1:** PK parameters of plasma total radioactivity and unlabeled SH-1028 following a single oral dose of 7.18 MBq (194 μCi)/15 mg/kg [^14^C]SH-1028 in rats (n = 6).

PK parameter (units)	Total radioactivity in plasma	Unlabeled SH-1028
	Mean ± SD	Mean ± SD
T_max_ (h)	4 ± 2	5 ± 2
C_max_ ^a^	694 ± 330	236 ± 170
t_1/2_ (h)	18.7 ± 2.2	2.8 ± 0.6
AUC_0-t_ ^a^	11917 ± 4670	1837 ± 1499
${\text{AUC}}_{0-\infty }$ ^a^	14374 ± 5380	2554 ± 1346
MRT (h)	25.5 ± 2.2	5.8 ± 0.9

^a^ ng eq./mL for C_max_ and h·ng eq./mL for AUC, for total radioactivity in plasma; ng/mL for C_max_ and h·ng/mL for AUC, for unlabeled SH-1028.

#### 3.1.2 Mass balance

After a single oral dose of 6.96 MBq (188 μCi)/14.6 mg/kg [^14^C]SH-1028 to rats, the cumulative recoveries of radioactivity in urine, feces and bile (bile duct-cannulated rats) are shown in Supplementary Figure S3. By 168 h post-dose, the cumulative excretion of radioactivity in urine and feces accounted for 86.0% of the administered dose, with 1.42% recovered in urine and 84.5% in feces. In bile duct-cannulated rats, 22.0% of the dose was recovered in bile within 48 h post-dose, indicating biliary excretion is the primary route for the elimination of drug-related materials, which is subsequently excreted in feces.

#### 3.1.3 Metabolite profiling and identification

In addition to the parent drug, a total of 38 metabolites were identified in rats. Metabolites were named in order of increasing mass-to-charge ratio (m/z). For metabolites with identical m/z values, naming followed increasing chromatographic retention time. The proposed metabolic pathways of SH-1028 in rats are illustrated in Figure 1.

##### 3.1.3.1 Metabolite profiling in plasma

Only the unchanged parent drug, SH-1028, was detected in rat plasma by radiochromatography. However, analysis by UPLC/Triple TOF MS revealed 13 additional metabolites, including demethylated metabolites (M525a/b[M3-1] and Imp3[M3-2]), demethylated and mono-oxygenated metabolites (M541 [M4-7]), mono-oxygenated metabolites (M555[M5-1, M5-2, M5-3, M5-4 and M5-6]), di-oxygenated metabolites (M571 [M6-1, M6-5]) and mono-oxygenated glucuronide conjugates (M731[M11-1, M11-2 and M11-3]).

##### 3.1.3.2 Metabolite profiling in urine

Drug-related material was minimally excreted in rat urine, accounting for merely 1.42% of the administered dose, suggesting that urinary metabolite concentrations are low. Only three relevant ions were detected in rat urine samples, with m/z values of 556.3 (M555 [M5-4]) and 732.3 (M731[M11-1 and M11-3]). Due to their extremely low concentrations, these metabolites were undetectable by radiochromatography.

##### 3.1.3.3 Metabolite profiling in feces and bile

SH-1028 underwent extensive metabolism in rats. The parent drug accounted for 2.53% and 9.29% of the total radiochromatographic peak area in feces from male and female rats, respectively, and 2.83% and 1.98% in bile. Apart from the parent drug, 28 metabolites were identified in feces and 35 in bile. In feces, M525a/b [M3-1] was the most abundant metabolite, accounting for 28.3% and 24.7% of peak area in males and females, respectively; all other metabolites accounted for less than 10%. In bile, M511 [M2] and M521a/b [M3-1] were the predominant metabolites, accounting for 8.69% and 7.95% of peak area in males, and 9.56% and 6.30% in females, respectively; all others were below 5%.

#### 3.1.4 Rat QWBA

**FIGURE 2:**
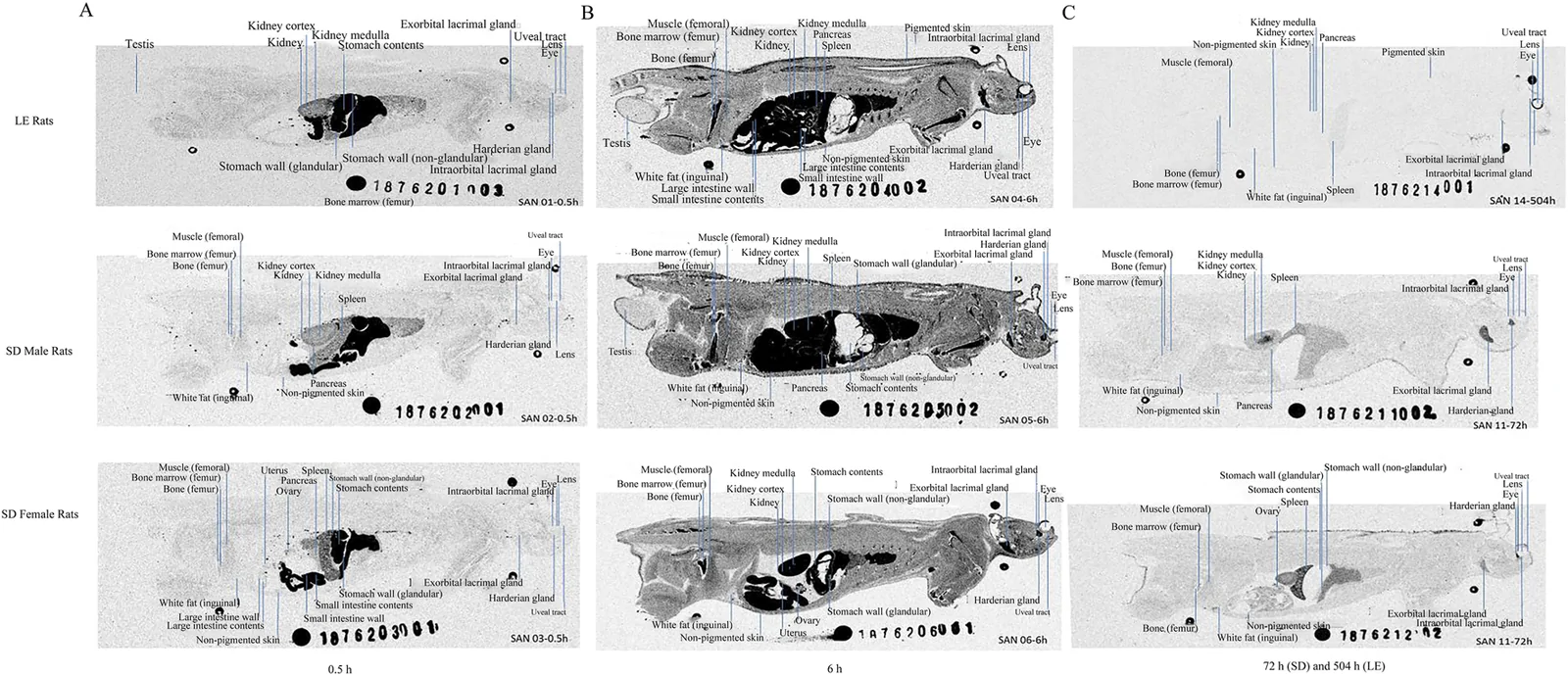
**(A–C)** Whole-body autoradiograms (QWBA) of selected cross-sections of LE rats, SD male and female rats at different time points.

Following a single oral dose of [^14^C]SH-1028 to rats, the tissue distribution of total radioactivity was assessed by QWBA across 47 tissues (Supplementary Figure S4). Total radioactivity in most tissues peaked at 0.5 or 6 h post-dose, with subsequent slow elimination. At 72 h (the last time point for SD rats), radioactivity remained detectable in the vast majority of tissues (Figure 2). The distribution and elimination profiles in pigmented LE rats were comparable to those observed in SD rats. At 504 h (the terminal time point for LE rats), radioactivity fell below the LLOQ in plasma, whole brain, cervical lymph nodes, small intestinal contents, spinal cord, non-glandular gastric wall, white adipose tissue and stomach contents; all other tissues, including intestinal contents, still exhibited detectable levels (Figure 2). Tissue-to-plasma AUC_0-t_ ratios revealed limited distribution to brain, spinal cord and white adipose tissue (ratios <1), with extensive distribution to all other tissues (ratios >>1). In LE rats, total radioactivity also distributed to pigment-associated tissues, peaking at 72 h in the uveal tract and 24 h in pigmented skin. At 504 h, total radioactivity in these tissues was approximately 89.67% and 2.83% of their respective C_max_ values, indicating reversible pigment binding with slow elimination of SH-1028 and its metabolites.

### 3.2 Human mass balance results

#### 3.2.1 Demographic characteristics

Four healthy male subjects were enrolled and completed the study according to protocol. All subjects were included in the safety and PK analysis. Each subject received a single oral dose of 200 mg SH-1028 containing approximately 3.26 MBq (88 μCi) of [^14^C]SH-1028 as an oral suspension. Their average age, weight and BMI were 32.8 years old (range 30–36 years), 66.73 kg (range 56.7–78.7 kg) and 22.7 kg/m^2^ (range 19.6–25.5 kg/m^2^), respectively (Table 2).

**TABLE 2:** Demographic characteristics of the subjects.

Characteristic	[^14^C]SH-1028 (N = 4)
Male sex, NO.	4
Han race, NO	4
Age, mean (range), years	32.8 (30, 36)
Height, mean (range), cm	171.18 (169.2, 176)
Weight, mean (range), kg	66.73 (56.7, 78.7)
BMI, mean (range), kg/m^2^	22.7 (19.6, 25.5)

#### 3.2.2 PK results

**FIGURE 3:**
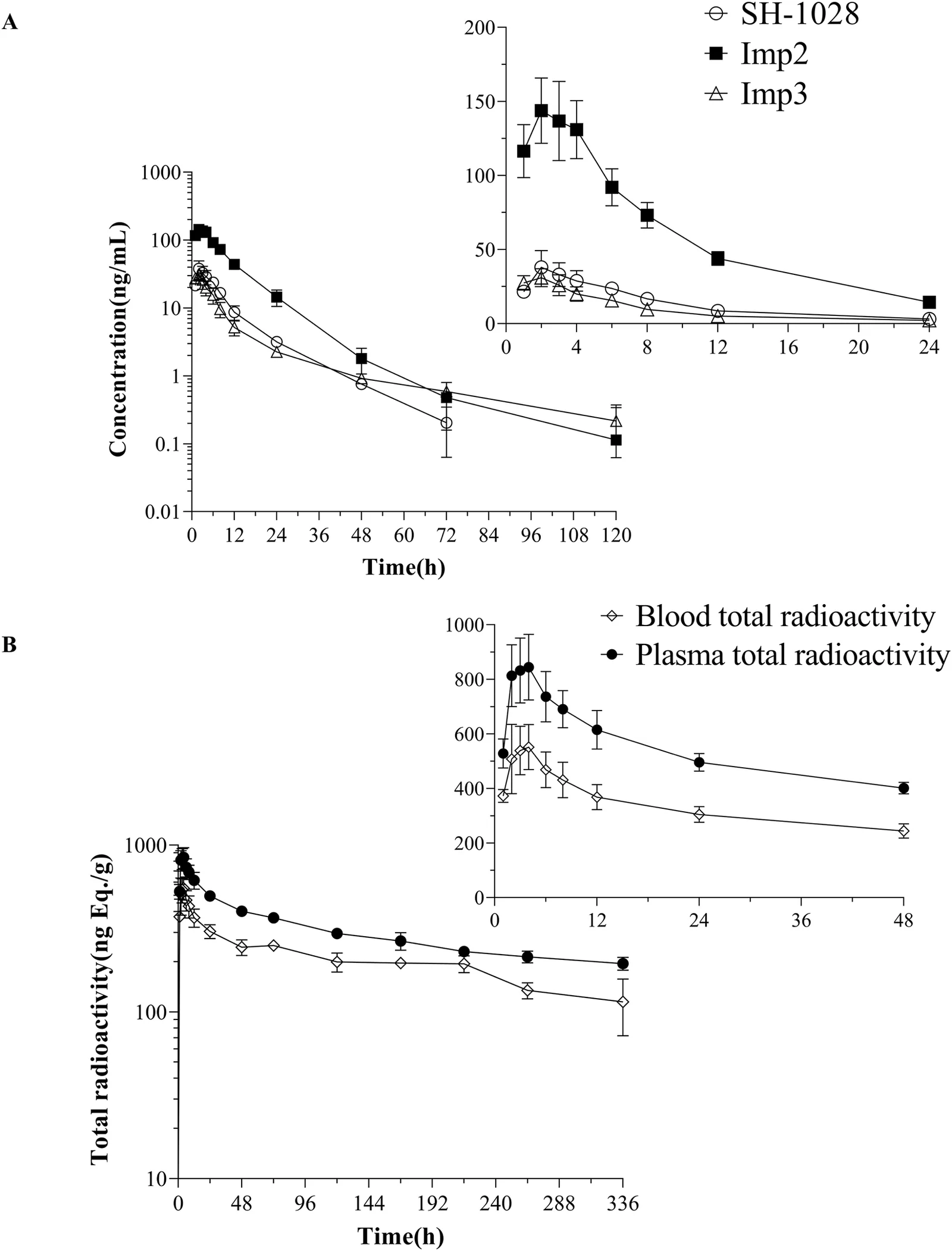
Mean (±SD) concentration-time profiles for SH-1028, its metabolites (Imp2 and Imp3), and total radioactivity following a single oral dose of 200 mg containing 3.26 MBq (88 μCi) of [^14^C]SH-1028 in healthy subjects (n = 4). **(A)** Mean plasma concentration-time profiles for SH-1028, Imp2 and Imp3. **(B)** Mean plasma and blood concentration-time profiles for total radioactivity.

The mean plasma concentration-time profiles for unlabeled SH-1028, Imp2 and Imp3 after a single oral dose of 200 mg [^14^C]SH-1028 are presented in Figure 3, with the corresponding PK parameters are summarized in Table 3. All four subjects showed consistent PK behavior. The parent drug SH-1028 and metabolite Imp3 reached their peak concentrations at 2.0 post-dose, whereas Imp2 peaked later at 2.5 h post-dose. The mean C_max_ for SH-1028, Imp2 and Imp3 were 38.8, 146.8 and 32.0 ng/mL, respectively, and the mean ${\text{AUC}}_{0-\infty }$ were 385.9, 1671.3 and 320.6 h ng/mL, respectively. The mean terminal elimination half-life (t_1/2_) of unlabeled SH-1028, Imp2 and Imp3 were 12.5, 9.6 and 38.4 h, respectively.

**TABLE 3:** PK parameters of unlabeled SH-1028, its metabolites (Imp2 and Imp3), and total radioactivity, following a single oral dose of 200 mg containing 3.26 MBq (88 μCi) of [^14^C]SH-1028 in healthy subjects (n = 4).

Parameter^a^(units)	SH-1028	Imp2	Imp3	Total radioactivity in plasma	Total radioactivity in whole blood
T_max_ (h)	2.0 (2, 3)	2.5 (2, 4)	2.0 (1, 2)	4.0 (4, 4)	4.0 (2, 4)
C_max_^b^	38.8 ± 11.4	146.8 ± 21.9	32.0 ± 5.1	844.8 ± 120.2	556.0 ± 88.7
t_1/2_ (h)	12.5 ± 1.2	9.6 ± 3.2	38.4 ± 15.5	511.3 ± 144.8	477.7 ± 211.8
AUC_0-t_^b^	379.2 ± 62.6	1663.2 ± 79.3	302.3 ± 64.7	186728.3 ± 25636.9	120527.7 ± 42827.4
${\text{AUC}}_{0-\infty }$ ^b^	385.9 ± 59.7	1671.3 ± 79.1	320.6 ± 68.1	248672.5 ± 38692.5	198197.5 ± 88192.5
CL/F (L/h)	522.6 ± 82.2	118.7 ± 5.7	639.3 ± 136.0	0.8 ± 0.1	1.1 ± 0.5
V_d_/F (L)	9390.6 ± 1635.5	1641.6 ± 516.2	34093.9 ± 12035.7	580.8 ± 100.3	686.7 ± 82.4
MRT_0-t_ (h)	11.1 ± 1.3	10.8 ± 1.9	17.0 ± 2.0	348.3 ± 82.5	315.4 ± 159.1
${\text{MRT}}_{0-\infty }$ (h)	12.4 ± 1.3	11.2 ± 2.0	25.5 ± 6.1	688.8 ± 189.1	761.0 ± 404.3

^a^ The data for all parameters were presented as mean ± SD except Tmax, which was presented as median (range).

^b^ ng/mL for Cmax and h·ng/mL for AUC, for SH-1028, Imp2 and Imp3; ng eq./g for Cmax and h·ng eq./g for AUC, for total radioactivity in plasma and whole blood.

The mean concentration-time profiles for total radioactivity in blood and plasma are shown in Figure 3, and the mean PK parameters are listed in Table 3. The total radioactivity in plasma and blood both reached C_max_ at 4.0 h (median T_max_), with mean C_max_ of 844.8 and 556.0 ng Eq./g, respectively. The total radioactivity was eliminated slowly, with mean t_1/2_ of 511.3 h in plasma and 477.7 h in blood. The plasma ${\text{AUC}}_{0-\infty }$ of unlabeled SH-1028, Imp2 and Imp3 accounted for approximately 0.16%, 0.67% and 0.13% of the mean total radioactivity ${\text{AUC}}_{0-\infty }$ (248672.5 h ng Eq./g) in plasma, respectively.

The ratio of total radioactivity concentration in whole blood to that in plasma across all measured time points ranged from 0.59 to 0.85 (Supplementary Figure S5), suggesting that there was no indication of a trend in the association of the radioactivity with the red blood cells.

#### 3.2.3 Mass balance

**FIGURE 4:**
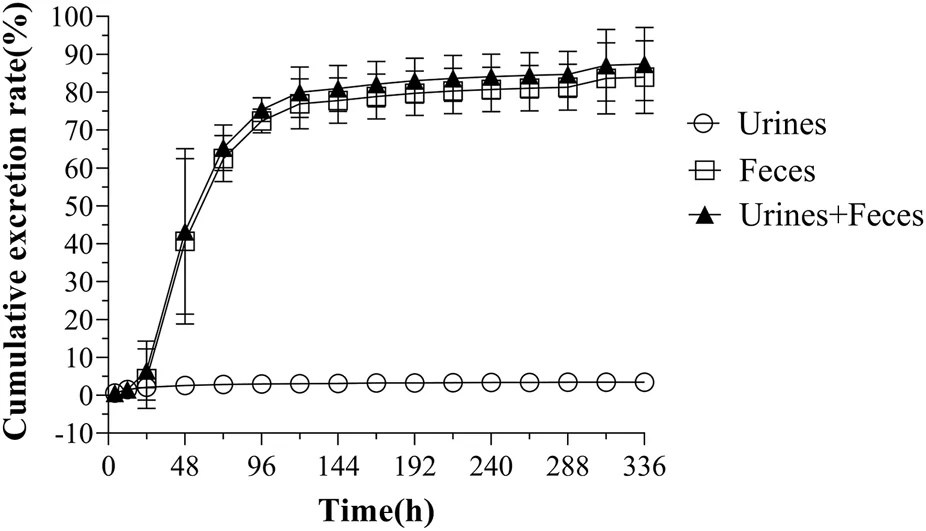
Mean (±SD) cumulative excretion of radioactivity in urine and feces following a single oral dose of 200 mg containing 3.26 MBq (88 μCi) of [^14^C]SH-1028 (n = 4).

After a single oral dose of 200 mg containing 3.26 MBq (88 μCi) of [^14^C]SH-1028 to four healthy subjects, the cumulative recoveries of radioactivity in urine and feces are shown in Figure 4. Near-complete recovery of administered radioactivity was achieved, with a mean of 85.02% of the administered dose recovered within 288 h or 336 h post-dose. The mean radioactivity recovered in urine and feces over the collection period was 3.48% (range, 3.38%–3.56%) and 81.55% (range, 77.18%–90.75%), respectively, indicating that fecal excretion was the predominant elimination route for drug-related material. Actually, within 120 h post-dose, the recovery of radioactivity had nearly reached a plateau phase, with an average recovery of 80.01%.

#### 3.2.4 Metabolite profiling and identification

**FIGURE 5:**
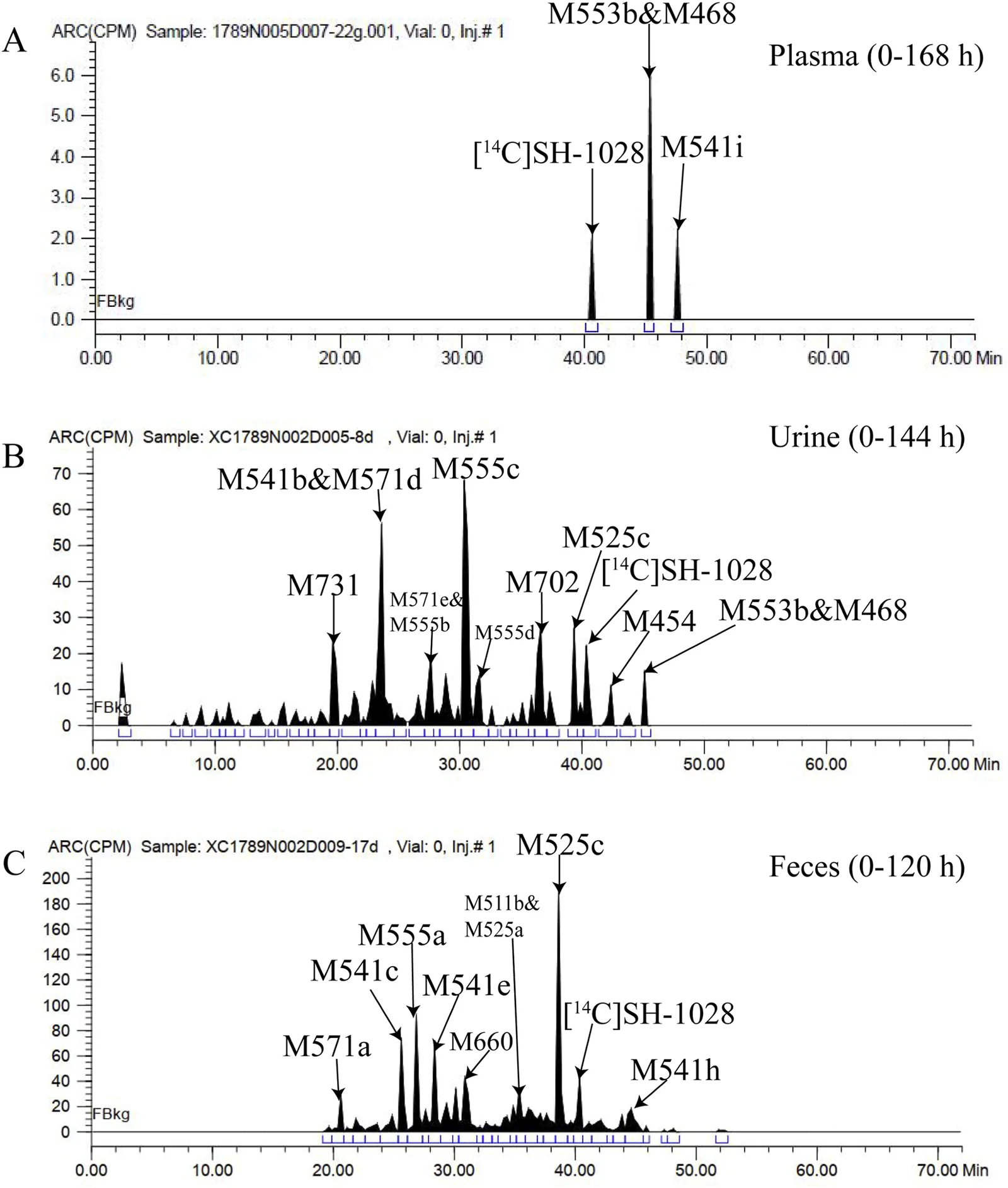
Representative HPLC-radiochromatograms from plasma, urine and feces across the four subjects. **(A)** The plasma chromatogram was generated from an AUC pooled sample of the four subjects (0–168 h); **(B)** The urine chromatogram was generated from a pool of the four subjects collected between 0 and 144 h post-dose; **(C)** The feces chromatogram was generated from a pool of the four subjects collected between 0 and 120 h post-dose.

The representative radio-HPLC profiles of pooled plasma, urine and fecal samples across the four subjects are depicted in Figure 5, and the metabolite profiling data are summarized in Table 4. Following a single oral dose of 200 mg containing 3.26 MBq (88 μCi) of [^14^C]SH-1028, a total of 14 metabolites were identified in the pooled plasma and 33 metabolites in the excreta samples. The primary metabolic pathways in humans were mono-oxidation and demethylation. The secondary metabolic pathways were cysteine conjugation and N-dealkylation. The proposed metabolic pathway of SH-1028 in humans is demonstrated in Figure 1.

**TABLE 4:** Summary of SH-1028 metabolite quantification and identification in pooled plasma, pooled urine (0–144 h) and pooled feces (0–120 h) after an oral dose of [^14^C]SH-1028 in humans.

Name	Molecular formula	[M+H]^+^ (m/z)	Plasma (%TRA)						Urine		Feces		Urine+Feces
​			2 h	4 h	8 h	12 h	48 h	0–168 h	TRA (%)	Dose (%)	TRA (%)	Dose (%)	Dose (%)
[^14^C]SH-1028	C_30_^14^CH_38_N_7_O_2_	542.3137	9.56	7.69	3.33	8.62	20.00	20.00	4.31	0.14	4.39	3.38	3.52
M717	C_36_H_44_N_7_O_9_	718.3182	−	−	−	−	−	−	1.21	0.04	ND	ND	0.04
M731	C_37_H_46_N_7_O_9_	732.3337	−	−	−	−	−	−	5.53	0.18	0.45	0.35	0.53
M571a	C_31_H_38_N_7_O_4_	572.2977	−	−	−	−	−	−	ND	ND	2.68	2.06	2.06
M571b	C_31_H_38_N_7_O_4_	572.2977	−	−	−	−	−	−	3.23	0.10	0.45	0.35	0.45
M541a	C_30_H_36_N_7_O_3_	542.2875	−	−	−	−	−	−	2.83	0.09	+	+	0.09
M571c	C_31_H_38_N_7_O_4_	572.2979	0.74	3.85	6.67	3.45	ND	ND	+	+	+	+	+
M541b, M571d	M541b: C_30_H_36_N_7_O_3_ M571d: C_31_H_38_N_7_O_4_	M541b: 542.2875 M571d: 572.2978	−	−	−	−	−	−	14.02	0.45	1.34	1.03	1.48
M541c	C_30_H_36_N_7_O_3_	542.2871	−	−	−	−	−	−	ND	ND	7.14	5.49	5.49
M541d	C_30_H_36_N_7_O_3_	542.2872	−	−	−	−	−	−	+	+	+	+	+
M555a	C_31_H_38_N_7_O_3_	556.3030	−	−	−	−	−	−	2.43	0.08	8.48	6.52	6.60
M571e, M555b	M571e: C_31_H_38_N_7_O_4_ M555b: C_31_H_38_N_7_O	M571e: 572.2977 M555b: 556.3030	1.47	ND	ND	ND	ND	ND	4.04	0.13	1.78	1.37	1.50
M541e	C_30_H_36_N_7_O_3_	542.2872	ND	ND	ND	1.72	ND	ND	0.94	0.03	7.14	5.49	5.52
M541f, M571f	M541f: C_30_H_36_N_7_O_3_ M571f: C_31_H_38_N_7_O_4_	M541f: 542.2870 M571f: 572.2977	−	−	−	−	−	−	0.81	0.03	3.94	3.03	3.06
M555c	C_31_H_38_N_7_O_3_	556.3027	3.68	ND	ND	ND	ND	ND	17.52	0.56	3.12	2.40	2.96
M660	C_34_H_45_N_8_O_4_S	661.3271	−	−	−	−	−	−	+	+	7.43	5.71	5.71
M555d	C_31_H_38_N_7_O_3_	556.3030	4.41	0.96	ND	ND	ND	ND	3.50	0.11	+	+	0.11
M553a, M511a	M553a: C_31_H_36_N_7_O_3_ M511a: C_29_H_34_N_7_O_2_	M553a: 554.2870 M511a: 512.2763	−	−	−	−	−	−	1.08	0.03	2.08	1.60	1.63
M511b, M525a	M511b: C_29_H_34_N_7_O_2_ M525a: C_30_H_36_N_7_O_2_	M511b: 512.2768 M525a: 526.2924	−	−	−	−	−	−	ND	ND	4.24	3.26	3.26
M525b	C_30_H_36_N_7_O_2_	526.2923	7.35	1.92	5.00	12.07	ND	ND	1.21	0.04	4.31	3.31	3.35
M702	C_36_H_47_N_8_O_5_S	703.3382	1.47	ND	ND	ND	ND	ND	7.01	0.22	1.64	1.26	1.48
M511c, M541g	M511c: C_29_H_34_N_7_O_2_ M541 g: C_30_H_36_N_7_O_3_	M511c: 512.2769 M541 g: 542.2874	−	−	−	−	−	−	1.89	0.06	2.83	2.18	2.24
M525c (Imp3)	C_30_H_36_N_7_O_2_	526.2921	5.88	2.88	5.00	3.45	ND	ND	3.91	0.12	17.40	13.38	13.5
M454	C_26_H_27_N_6_O_2_	455.2175	14.71	15.38	10.00	8.62	ND	ND	2.02	0.06	2.38	1.83	1.89
M541 h	C_30_H_36_N_7_O_3_	542.2872	1.47	ND	ND	ND	ND	ND	+	+	3.87	2.98	2.98
M553b, M468(Imp2)	M553b: C_31_H_36_N_7_O_3_ Imp2: C_27_H_29_N_6_O_2_	M553b: 554.2852 Imp2: 469.2328	44.12	43.27	36.67	34.48	ND	60.00	2.02	0.06	+	+	0.06
M541i	C_30_H_36_N_7_O_3_	542.2888	3.68	16.35	26.67	20.69	80.00	20.00	+	+	+	+	+
Total (%)	​	​	98.54	92.3	93.34	93.1	100	100	79.51	2.53	87.09	66.98	69.51

ND, not detectable; −, Only identified in urine and fecal samples, not detected in plasma samples. +, Only observed in mass spectrometry.

##### 3.2.4.1 Metabolite profiling in plasma

Plasma samples collected at 2, 4, 8, 12 and 48 h post-dose were pooled at equal volumes and analyzed. Unchanged SH-1028 (m/z 542) accounted for 9.56%, 7.69%, 3.33%, 8.62% and 20.00% of the total radioactivity (TRA), respectively. M553b&Imp2 (which could not be separated under the employed LC conditions), M541i, M454, M525b, Imp3, and M571c were the predominant analytes in plasma at each time point, accounting for 44.12%, 3.68%, 14.71%, 7.35%, 5.88% and 0.74% TRA at 2 h; 43.27%, 16.35%, 15.38%, 1.92%, 2.88% and 3.85% TRA at 4 h; 36.67%, 26.67%, 10.00%, 5.00%, 5.00%, and 6.67% TRA at 8 h; 34.48%, 20.69%, 8.62%, 12.07%, 3.45% and 3.45% TRA at 12 h; and 0.00%, 80.00%, 0.00%, 0.00%, 0.00%, and 0.00% TRA at 48 h, respectively. Other circulating metabolites accounted for less than 5% TRA at each time point.

In the AUC-pooled 0–168 h plasma sample, only unchanged SH-1028, metabolites M553b and Imp2 and M541i were detected, accounting for 20.00%, 60.00% and 20.00% TRA, respectively.

##### 3.2.4.2 Metabolite profiling in urine

Excretion of drug-related material in urine was minimal, accounting for only 3.48% of the administered dose. Metabolite profiling of the 0–144 h human urine sample pooled by equal volume (accounting for 3.18% of the administered dose) revealed that unchanged SH-1028 represented only 0.14% of the dose, while the major metabolites were M555c, M541b&M571d (which not separated under the employed LC conditions), M702 and M731, which accounting for 0.56%, 0.45%, 0.22% and 0.18% of the dose, respectively. The other metabolites were minor components in urine, each accounting for less than 5% TRA and less than 0.15% of the administered dose. Collectively, the identified drug-related material represented 80% of the total radioactivity in urine.

##### 3.2.4.3 Metabolite profiling in feces

The fecal excretion was the predominant elimination route for drug-related radioactivity, accounting for 81.55% of the administered dose. Metabolite profiling of the pooled 0–120 h fecal sample (representing 76.91% of the administered dose) revealed that unchanged SH-1028 accounted for only 3.38% of the dose, while the major metabolites were Imp3, M555a, M660, M541c and M541e, which accounting for 13.38%, 6.52%, 5.71%, 5.49% and 5.49% of the dose, respectively. The other metabolites were minor components in feces, accounting for less than 5% TRA and less than 3.5% of the administered dose. Overall, unchanged SH-1028 and the identified metabolites represented 87.09% of the total radioactivity in feces.

#### 3.2.5 Safety and tolerability

SH-1028 was safe and well tolerated following a single oral administration of 200 mg containing 3.26 MBq (88 μCi) of [^14^C]SH-1028. A total of 8 AEs were reported in two subjects. Five of these AEs, occurring in two subjects, were assessed as treatment-related. These consisted of increased γ-glutamyl transferase (2 events in two subjects) and increased alanine aminotransferase (3 events in one subject). No serious adverse events (SAEs) were reported, and no AEs led to subject discontinuation. All reported AEs were mild (Grade 1) in severity and resolved spontaneously or completely recovered after intervention. Most AEs were laboratory abnormalities without clinical symptoms, except for one case of oral mucositis. No clinically significant abnormalities or changes in vital signs, electrocardiogram, or physical examinations were observed throughout the study.

## 4 Discussion

SH-1028 is a novel, irreversible third-generation EGFR-TKI developed for the treatment of locally advanced or metastatic NSCLC with EGFR T790M mutation-positive. Radiolabeling with the isotope ^14^C was employed to conduct an integrated preclinical and clinical investigation, encompassing mass balance studies in rats, QWBA, and a human mass balance study. These studies systematically elucidated the disposition of the drug *in vivo*, and clarified the rate, extent and form of exposure to SH-1028 and related substances. They also defined the major excretion pathways and the extent of elimination. These findings provide a robust PK foundation to support subsequent research and the clinical application of SH-1028. Based on the ethical requirements for radiolabeled studies and the principle of minimizing unnecessary radiation exposure, the human mass balance study was conducted with four subjects. This sample size that proved adequate to meet the predefined study objectives.

In a radiolabeled human mass balance study, two doses are typically considered: the pharmacological dose and the radioactive dose (Nijenhuis et al., 2016). The selected pharmacological dose of 200 mg was selected based on prior phase I studies in both patients and healthy subjects. These studies demonstrated favorable safety, tolerability, and pharmacokinetics at this dose level (He et al., 2023). This selected dose aligns with the clinically approved therapeutic dose. The radioactive dose was determined following a radiation dose estimate based on preclinical tissue QWBA and excretion data in rats (ICRP Publication 53, 1988). After oral administration of 3.7 MBq (100 μCi) of [^14^C]SH-1028, the calculated total effective dose (ED) in subjects was approximately 0.553 mSv. This value is much lower than the single dose limit of 30 mSv for adult research presented in 21 CFR 361.1 (Food and Drug Administration and HHS, 2002). However, the estimated radiation dose to the uvea per kilogram (56.7 mSv) slightly exceeded the 50 mSv limit for other tissues. Based on this limit, the calculated radioactive administered dose should be 3.26 MBq (88 μCi) per subject. When the total ED from whole-body radiation is less than 1 mSv, it would be in Risk Category IIb according to International Commission on Radiologic Protection (ICRP) 62. Therefore, a radioactive dose of 3.26 MBq (88 μCi) was ultimately adopted for the human mass balance.

The key PK parameters of unlabeled SH-1028 and its metabolites Imp2 and Imp3 observed in this study were consistent with those reported in a previous DDI study (Li et al., 2023). Following a single oral administration of 200 mg containing 3.26 MBq (88 μCi) of [^14^C]SH-1028, the total radioactivity in plasma peaked relatively quickly, with a median T_max_ of 4.0 h, which was slightly later than the T_max_ of unlabeled SH-1028 (2.3 h). Notably, the plasma total exposure of unlabeled SH-1028 and its metabolites Imp2 and Imp3 accounted for less than 1% of the plasma total radioactivity exposure. The mean terminal elimination t_1/2_ of total radioactivity in plasma was 511.3 h, which was significantly longer than that of SH-1028 (12.5 h), Imp2 (9.6 h) and Imp3 (38.4 h). One possible explanation for the prolonged terminal elimination t_1/2_ of total radioactivity is the formation of additional metabolites with slower systemic clearance; alternatively, sustained retention of drug-related material in the body may contribute to the extended terminal phase. Consistent with this interpretation, QWBA in LE rats demonstrated widespread tissue distribution of radioactivity, with detectable levels persisting in most tissues up to 504 h post-dose, suggesting slow redistribution of drug-related material from tissue compartments into the systemic circulation. This indicates the presence of a tissue reservoir that may contribute to the prolonged elimination phase of total radioactivity. Furthermore, the prolonged elimination t_1/2_ may be attributed to covalent binding to plasma proteins, given that the observed half-life (511 h) falls within the range of albumin turnover (approximately 19 days), suggesting that a fraction of drug-related radioactivity is associated with plasma proteins or other slow-turnover biological matrices. Notably, a similar phenomenon has been observed with osimertinib, for which the mean terminal t_1/2_ values of the parent drug and its metabolites AZ5104 and AZ7550 are 61.2, 55.2, and 82.0 h, respectively, whereas that for total plasma radioactivity is 474 h (Dickinson et al., 2016). Experimental confirmation of the putative covalent protein adducts (e.g., via ^14^C-protein specific radioanalysis) may be considered in future studies to better understand the nature of these associations.

The excretion profile of SH-1028 was characterized by predominant fecal elimination. In humans, a mean of 85.02% of the administered radioactivity was recovered over a 288–336 h collection period, with 81.55% excreted in feces and only 3.48% in urine. This pattern is consistent with the rat mass balance results (84.5% in feces, 1.42% in urine), confirming the adequacy of the sample collection period in the clinical study. Meanwhile, the low proportion of renal excretion suggests that dose adjustment may not be necessary for patients with renal impairment. Metabolite profiling revealed that unchanged SH-1028 accounted for only 3.38% of the administered dose in feces and 0.14% in urine, indicating that SH-1028 underwent extensive metabolic transformation *in vivo* and was primarily eliminated as metabolites via feces. Accordingly, unabsorbed parent drug is considered to contribute minimally to the overall fecal radioactivity. The bile duct-cannulated rat study showed that approximately 22% of the administered radioactivity was recovered in bile, supporting hepatobiliary excretion as an important elimination pathway for drug-related materials. Given that SH-1028 is primarily metabolized by CYP3A4 and that CYP3A enzymes are expressed in both the liver and intestine, both organs may contribute to its overall biotransformation. Furthermore, intestinal metabolism and/or intestinal processing of drug-related materials may also contribute to fecal elimination. Collectively, these findings suggest that fecal radioactivity mainly reflects metabolites eliminated via the hepatobiliary route, with possible contributions from intestinal metabolism and enterohepatic recycling. Thus, clinical pharmacology studies in patients with hepatic impairment may be warranted to further assess the impact of reduced hepatic function on the systemic exposure of SH-1028 and its metabolites.

A total of 33 metabolites were identified in human plasma, urine and feces in this study. Two high-proportion metabolites were identified in human circulation system, namely, M541i and M553b&Imp2, accounting for 20% and 60% of the plasma total radioactivity, respectively. The radioactivity exposure of M553b&Imp2 was 3-fold higher than that of the parent drug (20.00%). Combined with the plasma PK results of unlabeled SH-1028 and Imp2, the plasma exposure of Imp2 (AUC_0-t_ of 1663.2 h ng/mL) was approximately 4-fold higher than that of SH-1028 (AUC_0-t_ of 379.2 h ng/mL), suggesting that the radioactive contribution in M553b&Imp2 was almost entirely derived from Imp2, indicating that Imp2 is a main metabolite in human plasma. For Imp2, quantification was performed in the phase I dose-escalation study (60–400 mg, covering and exceeding the recommended clinical dose of 200 mg once daily, NCT03603262), indicating that systemic exposure to Imp2 has been assessed across clinically relevant and supra-therapeutic dose levels, with no emerging safety concerns based on available evidence. For M541i, although its human exposure has been confirmed, direct clinical exposure-safety correlation data remain limited at present. As a major circulating metabolite exceeding the 10% threshold of total drug-related exposure (U.S. Food and Drug Ad ministration, 2020), the safety profile will require further evaluation in ongoing and future experiments. Importantly, SH-1028 has been approved and marketed in China since 2024 for the treatment of patients with locally advanced or metastatic NSCLC harboring *EGFR* T790M mutations. A multicenter phase II clinical study (n = 286) demonstrated that SH-1028 exhibited clinically meaningful antitumor activity with a manageable safety profile and no new or unexpected safety signals (Xiong et al., 2022), further supporting its overall tolerability in the intended patient population.

Interspecies differences in metabolic profiles were observed between rats and humans following administration of SH-1028. In rats, a total of 38 metabolites were identified in plasma and excreta samples. The parent compound SH-1028 was the predominant drug-related component in plasma radiochromatograms, and no circulating metabolites corresponding to the major human metabolites Imp2 and M541i were observed. In rat excreta, multiple metabolites were detected, with M525a/b identified as the predominant fecal metabolites, accounting for 28.3% and 24.7% of the administered dose in female and male rats, respectively. In contrast, fecal analysis in humans revealed Imp3 as the major metabolite, accounting for 17.40% of total radioactivity, indicating distinct metabolite excretion patterns between species. In humans, the primary metabolic pathways are mono-oxidation and demethylation, with secondary metabolic pathways including cysteine conjugation and N-dealkylation. In rats, however, the major metabolic pathways consist of demethylation, mono- and di-oxidation, and glucuronidation, as well as downstream metabolites formed via glutathione conjugation followed by hydrolysis, such as cysteinylglycine, cysteine and N-acetylcysteine conjugates. Collectively, these findings suggest qualitative and quantitative differences in metabolic pathways between rats and humans, with species-dependent formation and elimination of major metabolites. Accordingly, these interspecies metabolic differences warrant further clinical monitoring of the safety profiles of the major circulating human metabolites in ongoing and future clinical trials.

The QWBA study revealed limited distribution of SH-1028-related radioactivity to the central nervous system, with tissue-to-plasma AUC ratios below one in both brain and spinal cord, indicating restricted penetration of total drug-related material across the blood-brain barrier. However, QWBA reflects total radioactivity rather than unbound parent drug, and therefore does not directly represent the pharmacologically active fraction in brain tissue. CNS exposure of EGFR-TKIs is influenced by multiple factors, including efflux transporters, tissue binding, and unbound drug concentrations, which are not fully captured by total radioactivity measurements. Thus, limited brain distribution in QWBA does not necessarily indicate absence of pharmacological CNS activity. Indeed, previous phase II clinical data of SH-1028 (Xiong et al., 2022) demonstrated intracranial activity, with an intracranial objective response rate (iORR) of 35.5% and disease control rate (iDCR) of 93.5%. These values, however, appeared generally lower than those reported for other third-generation EGFR-TKIs with higher CNS exposure, such as osimertinib, which has shown an iORR of up to 84.2% and an iDCR of up to 94.7% in patients with asymptomatic brain metastases (Peled et al., 2021). In addition, PET imaging studies using ^11^C-labeled EGFR-TKIs (e.g., osimertinib) (Varrone et al., 2020) have provided more direct assessment of brain penetration and may offer complementary insights for future evaluation of SH-1028.

This study has several limitations. First, although this study was conducted between November 2020 and March 2021, current regulatory guidelines from the U.S. Food and Drug Administration (FDA) and the Center for Drug Evaluation of the National Medical Products Administration (CDE, NMPA) (U.S. Food and Drug Administration, 2024; Center for Drug Evaluation and National Medical Products Administration, 2024) generally recommend a minimum of six evaluable subjects for radiolabeled human mass balance studies to adequately characterize mass balance and metabolite profiles. In the present study, four healthy male subjects were completed, which is below the recommended sample size. Second, only male subjects were included in this radiolabeled mass balance study to ensure standardized and reliable urine and feces collection under controlled conditions; therefore, potential sex-related pharmacokinetic differences cannot be excluded and may warrant further evaluation if clinically relevant. Third, interspecies differences in metabolite profiles were observed between rats and humans; specifically, the presence of human-specific circulating metabolites Imp2 and M541i absent in rodents may reflect species-dependent metabolic pathways and may warrant further safety evaluation in subsequent studies. Despite these limitations and findings, the study achieved its primary objectives of characterizing mass balance, defining elimination pathways, and identifying major circulating and excreted metabolites.

Future studies may further explore the relationship between total and unbound central nervous system exposure of SH-1028, as QWBA suggests limited brain distribution despite clinically observed intracranial activity. Moreover, radiolabeled absolute bioavailability studies (via both intravenous and oral administration) may also be warranted to further elucidate the absorption and disposition profile of SH-1028. In addition, given the predominantly CYP3A-mediated metabolism and hepatobiliary elimination of SH-1028, as well as the presence of human-specific circulating metabolites, the potential influence of hepatic function and metabolite safety may warrant further consideration to better understand the clinical pharmacokinetics and safety profile of SH-1028.

## 5 Conclusion

This study systematically elucidated the metabolic transformation and elimination pathways of SH-1028 in rats and humans. Following a single oral dose of [^14^C]SH-1028, healthy subjects demonstrated good safety and tolerability. Two major metabolites, M541i and Imp2, were identified in human circulatory system, accounting for 20% and 60% of total plasma radioactivity, respectively. Excretion of drug-related substances occurred predominantly via feces in both rats and humans. The excretion of the parent drug in urine and feces accounted for only 3.52% of the administered dose, indicating that SH-1028 undergoes extensive metabolism *in vivo* and is eliminated predominantly in the form of metabolites.

These integrated preclinical and clinical data provide a comprehensive PK foundation for the rational clinical use of SH-1028 and inform future research on its metabolism and disposition.

## Data Availability

The original contributions presented in the study are included in the article/Supplementary Material, further inquiries can be directed to the corresponding author.
