# Supplementary material for: Absorption, distribution, metabolism, and excretion of [14C]SH-1028, a third-generation EGFR-TKI, in rats and humans
Source: Front Pharmacol. 2026 Aug 12;17:1897957. doi: 10.3389/fphar.2026.1897957 (PMC13506953; doi:10.3389/fphar.2026.1897957)
Supplement: Supplementary file 1 [file DataSheet1.pdf]

Absorption, Distribution, Metabolism, and Excretion of [^{14}C]SH-1028

Supplementary Materials

1. Rat ADME Study

The PK study was conducted in six SD rats (three males and three females). Each animal received a single oral dose of 7.18 MBq (194 μCi)/15.0 mg/kg [^{14}C]SH-1028. The rats were fasted overnight with free access to water prior to dosing. They were allowed access to food 2 h post-dose. Venous blood samples (approximately 0.3 mL) were collected before dosing and at 1, 2, 4, 6, 8, 24 and 48 h post-dose into heparinized tubes. The samples were centrifuged at 11,000 rpm for 5 min to separate the supernatant plasma samples, which were then stored at -20°C for analysis.

The mass balance study was conducted in twelve SD rats (six males and six females). Each animal received a single oral dose of 6.96 MBq (188 μCi)/14.6 mg/kg [^{14}C]SH-1028. Six rats were selected for urine and feces collection. After overnight fasting with free access to water, they were placed in metabolic cages for blank sample collection, followed by administration. Urine and feces were collected separately over the intervals of 0-4, 4-8, 8-24, 24-48, 48-72, 72-96, 96-120, 120-144, and 144-168 h post-dose. Urine samples were rinsed with 10 mL water, pooled and measured for volume. Fecal samples were weighed. Additionally, six rats were used for collection of bile samples. Following overnight fasting with free access to water, bile duct cannulation surgery was performed. Bile samples were collected at pre-dose and over the intervals of 0-2, 2-4, 4-8, 8-24, and 24-48 h post-dose. The volume was recorded. All samples were stored at -20°C.

The QWBA study was conducted in seven male Long-Evans (LE) rats and ten SD rats (five males and five females). Each rat received a single oral dose of [^{14}C]SH-1028 at 3.7 MBq (100 μCi)/15 mg/kg. At 0.5, 6, 24, 72, 168 (LE rats only), and 504 h (LE rats only) post-dose, rats were anesthetized with carbon dioxide and approximately 4 mL of whole blood samples were collected via cardiac puncture into EDTA-K_2 anticoagulant tubes. The selection of sampling time points was based on preliminary

nonclinical PK data to capture the early distribution and elimination phases, while extended time points in LE rats were included to evaluate potential long-term retention in melanin-containing tissues. Plasma samples were obtained by centrifugation at 2400 × g for 5 min at 4°C. Immediately after blood collection, carcasses were frozen in a dry ice bath containing n-hexane. All samples were stored at -20°C.

Determination of unlabeled SH-1028 in Plasma

Plasma concentrations of unlabeled SH-1028 were determined using a validated liquid chromatography-tandem mass spectrometry (LC-MS/MS) method. The LC-MS/MS system was conducted with a Sciex Triple Quad 6500 tandem MS (Applied Biosystems, CA, USA) coupled with either a 30-AD XR HPLC system (Shimadzu Co., Kyoto, Japan). For sample preparation, 25.0 μL of plasma was mixed with 25.0 μL of internal standard (IS), 25.0 μL of methanol-water (1:1, v/v), and 100 μL of acetonitrile to precipitate proteins. After vortexing for 1 min and centrifugation at 14,000 rpm for 5 min, 2.00 μL of the supernatant was injected for LC-MS/MS analysis.

The analytes were chromatographed on an YMC C18 column (5 μm， 50×2.0 mm) under gradient conditions with a flow rate of 0.5 mL/min. The mobile phases consisted of A (5 mM ammonium formate containing 0.2% formic acid) and B (acetonitrile). The ionization was conducted in positive ion mode using the selected transitions m/z 540.2 →495 for SH-1028 and m/z 543.1→470.5 for IS. The calibration range was from 3.00 ng/mL to 500 ng/mL, with the lower limit of quantitation of 3.00 ng/mL.

Measurement of Total Radioactivity

Total radioactivity in plasma, urine, feces and bile samples were measured by liquid scintillation counter (LSC). For liquid samples, aliquots of 50 μL of plasma, 100 μL of urine, or 25 μL of bile were directly mixed with 5 mL of scintillation cocktail. Fecal samples were homogenized with water, and approximately 0.3 g of the homogenate was accurately weighed and combusted using an OX-501 oxidizer (R.J. Harvey Instrument Corporation, Tappan, NY, USA). The released $^{14}CO_2$ was trapped in 15 mL of ^{14}C-specific scintillation cocktail for subsequent counting.

Metabolite Profiling and Identification

Plasma samples collected from each rat at pre-dose and at 2, 4 and 8 h post-dose

were pooled by gender and time point. An aliquot of 600 μL of the pooled plasma sample was mixed with 1800 μL of acetonitrile, vortexed for 1 min, and centrifuged at 11,000 rpm for 5 min. The entire supernatant was evaporated to dryness under a stream of nitrogen at 40°C. The residue was reconstituted in 300 μL of acetonitrile-water (50:50, v/v). Then, 90 μL of the reconstituted solution was used for HPLC radiochromatography analysis, and 7 μL was used for UPLC/Triple TOF MS analysis.

Urine samples collected from rats at pre-dose and post-dose were pooled according to gender and the volume of urine collected at each time interval to obtain pooled pre-dose and 0-24 h post-dose urine samples. After centrifugation at 11,000 rpm for 5 min, aliquots of 90 μL and 7 μL of the supernatant were subjected to HPLC-radiochromatographic analysis and UPLC/TripleTOF MS analysis, respectively.

Fecal homogenate samples collected from rats at pre-dose and post-dose were pooled according to gender and the weight of the homogenate at each time interval to obtain pooled pre-dose and 0-48 h post-dose fecal homogenates. To the pooled sample, 2 mL of acetonitrile was added, vortexed for 1 min, ultrasonicated for 10 min, and centrifuged at 3,500 rpm for 5 min. The entire supernatant was transferred. The residue was extracted once more with 2 mL of acetonitrile-water (50:50, v/v). All supernatants were combined and evaporated to dryness under nitrogen at 40°C. The residue was reconstituted in 1000 μL of acetonitrile-water (50:50, v/v). An 80 μL aliquot of the supernatant was used for HPLC radiochromatography analysis, and a 7 μL aliquot was used for UPLC/Triple TOF MS analysis.

Bile samples collected from rats at pre-dose and post-dose were pooled according to gender and the volume of bile collected at each time interval to obtain pooled pre-dose and 0-48 h post-dose bile samples. After centrifugation at 11,000 rpm for 5 min, aliquots of 80 μL and 7 μL of the supernatant were subjected to HPLC-radiochromatographic analysis and UPLC/TripleTOF MS analysis, respectively.

Samples were analyzed with Agilent 1100 system on a column of Venusil C18 column (250×4.6mm, 5μm) under 25ºC temperature with mobile phase A (5 mM ammonium formate solution containing 0.2% formic acid) and B (acetonitrile) at the

flow rate of 0.6 mL/min. The gradient started as 20→30%B from 0 to 45min, 30-90% B from 45 to 55min and 90% B from 55 to 65min. Meanwhile, metabolites were identified using the Acquity UPLC liquid chromatography system coupled with the Triple TOF MS system.

Rat QWBA

For QWBA analysis, whole-body sagittal sections (30 μm thickness) were prepared from carcasses embedded in 2.5% carboxymethylcellulose (CMC), which included embedded quality control samples. Two adjacent sections were prepared for each anatomical region of interest. Following dehydration and labeling, the sections were co-exposed with pre-conditioned imaging plates at room temperature for 3 days. The exposed plates were then scanned using an Amersham Typhoon IP imager to obtain autoradiographic images. Radioactivity concentrations in selected tissues and regions were quantified by region-of-interest analysis using AIDA software (version 5.1; Raytest Isotopenmessgeräte GmbH, Straubenhardt, Germany).

In accordance with standard QWBA study design, a total of 47 tissues and organs of interest, including major ADME-related organs, barrier tissues, and melanin-containing tissues, were systematically selected for quantitative analysis to ensure comprehensive characterization of whole-body distribution and tissue retention.

The photostimulated luminescence intensity (PSL/mm^2) of tissues, standards and quality control (QC) samples was measured using AIDA. A standard curve was generated by plotting PSL/mm^2 (y-axis) against corresponding radioactivity concentrations (determined by combustion oxidation, x-axis), with linear regression performed using $1/x^2$ weighting factors. Tissue radioactivity concentrations were subsequently calculated from these calibration curves. Concurrently, the ratio of measured to labeled values (oxidizer combustion values) for QC samples from the tissue sections served as a section correction factor. Tissue radioactivity concentrations calculated from the standard curve were multiplied by this correction factor to obtain corrected actual concentrations. The standard curve was established using 10 different concentrations of ^{14}C standards ranging from approximately 18.5 to 75850 Bq/g. QC samples were prepared at low (67.71 Bq/g), medium (2009.1 Bq/g) and high (53835

Bq/g) concentration levels.

2. Detailed inclusion and exclusion criteria of subjects.

Inclusion criteria	Exclusion criteria
1. Healthy adult males, aged 18–50 years (inclusive);	1. Abnormal nervous system, respiratory system, cardiovascular system, digestive system, blood and lymphatic system, endocrine system or skeletal muscle system with clinical significance as determined by the investigator;
2. Body weight of ≥ 50 kg and body mass index (BMI) of 19.0–26.0 (inclusive);	2. Abnormal ECG corrected QT interval in resting state; various clinically significant abnormalities in heart rhythm, conduction and resting ECG morphology; various factors that may increase the risk of QTc prolongation or arrhythmia events;
3. Normal physical examination, vital signs, ECG, hematology, urinalysis, liver function, renal function, electrolytes, blood glucose and other laboratory tests, or mild abnormalities without clinical significance by the investigator;	3. Allergic constitution: those who are allergic to drugs, food, pollen, etc., and have a history of allergy to the active ingredient or excipients of the study drug;
4. No addiction to tobacco or alcohol, and agreement to refrain from tobacco, alcohol and caffeinated beverages during the study;	4. Habitual intake, and informed consent not to discontinue intake of any food or beverage containing alcohol, xanthine, or grapefruit, as well as chocolate, tea, coffee, or cola, which may affect drug absorption, distribution, metabolism, and excretion, until the end of the trial;
5. Fully understand the purpose, requirements, content, process, and possible adverse reactions of this trial, voluntarily participate in the trial, be able to complete the entire trial in accordance with the trial requirements and comply with the research regulations, and obtain informed consent in accordance with GCP regulations.	5. Having participated in any other clinical studies within 3 months before screening;
6. Participants and their spouses who have no plans to conceive within 6 months of the last administration of the study drug and who agree to use effective contraceptive measures.	6. Chronic constipation or diarrhea, irritable bowel syndrome, inflammatory bowel disease and/or perianal disease, or positive stool occult blood test in screening/at baseline;
	7. Workers who need to be exposed to radioactivity conditions for a long time or have significant radiation exposure within 1 year before the study (more than one radiation exposure from chest X-ray, CT scan or barium meal examination), or have participated in a radiolabeled drug study, or plan to receive high-level radiation within 12 months after the administration of the study drug.

3. Supplementary Figures

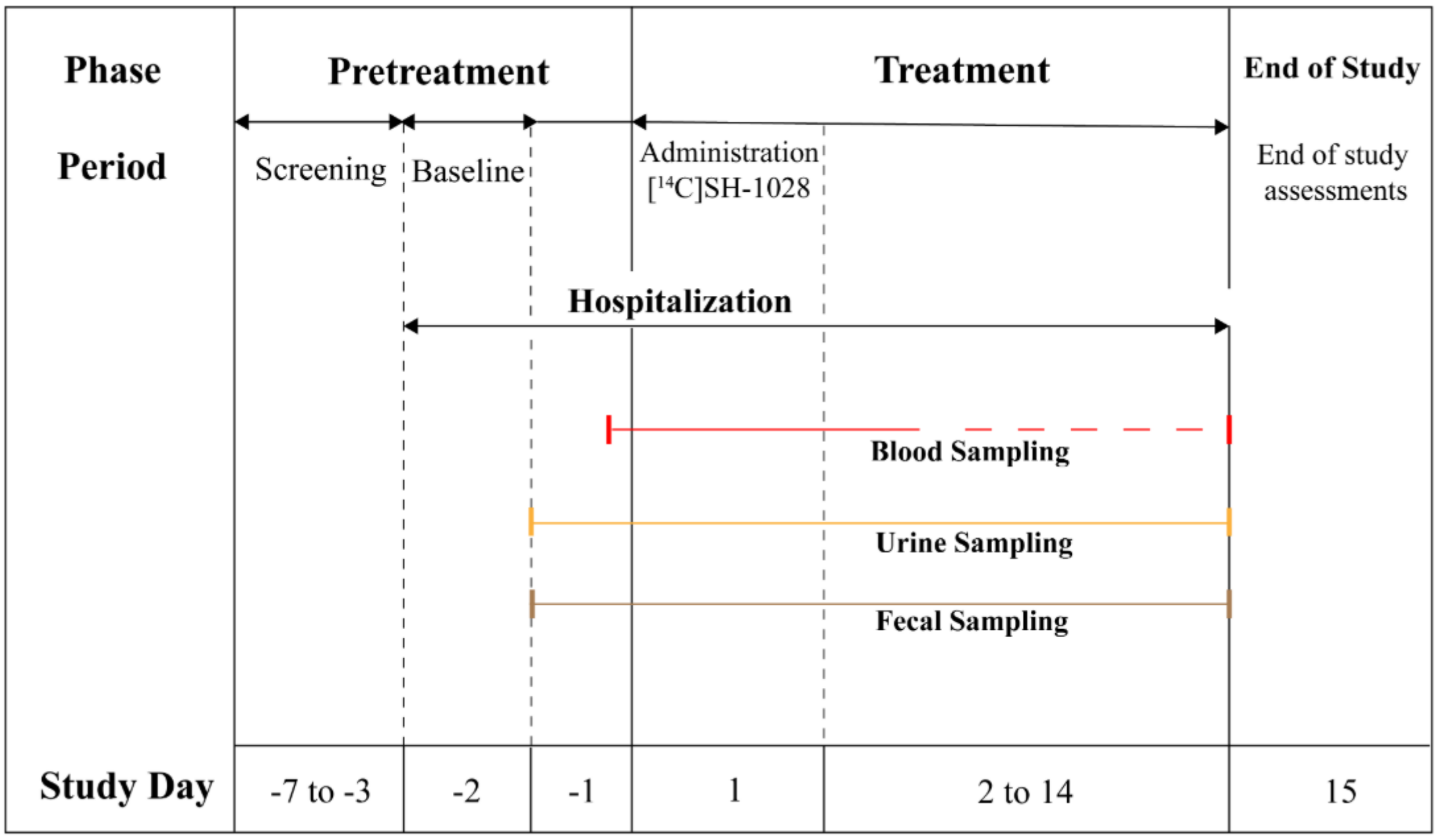

Supplementary Fig. 1 Schematic of clinical study design.

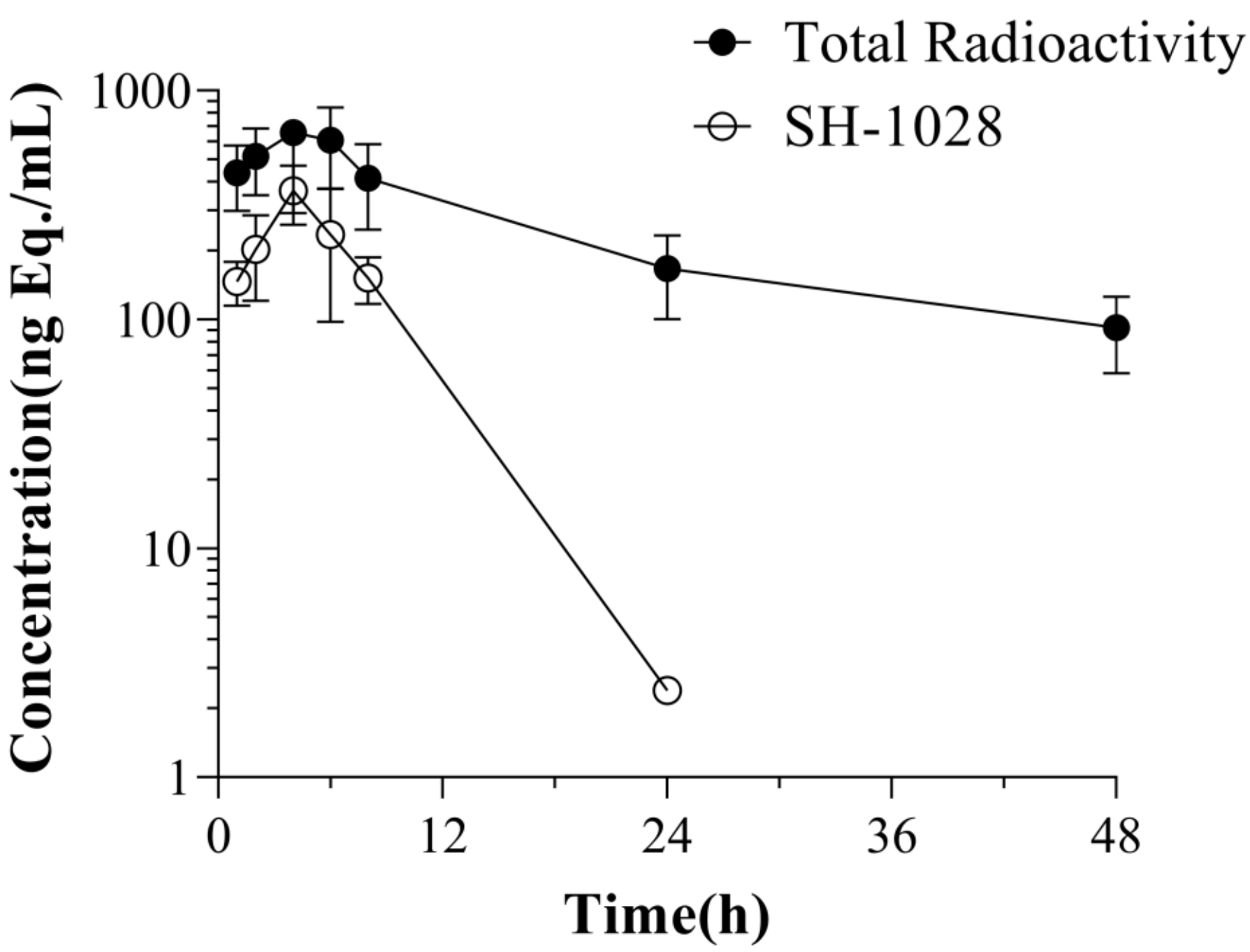

Supplementary Fig. 2 Mean (±SD) plasma concentration-time profiles for SH-1028 and total radioactivity following a single oral dose of 7.18 MBq (194 μCi)/15.0 mg/kg [^{14}C]SH-1028 in SD rats (n=6).

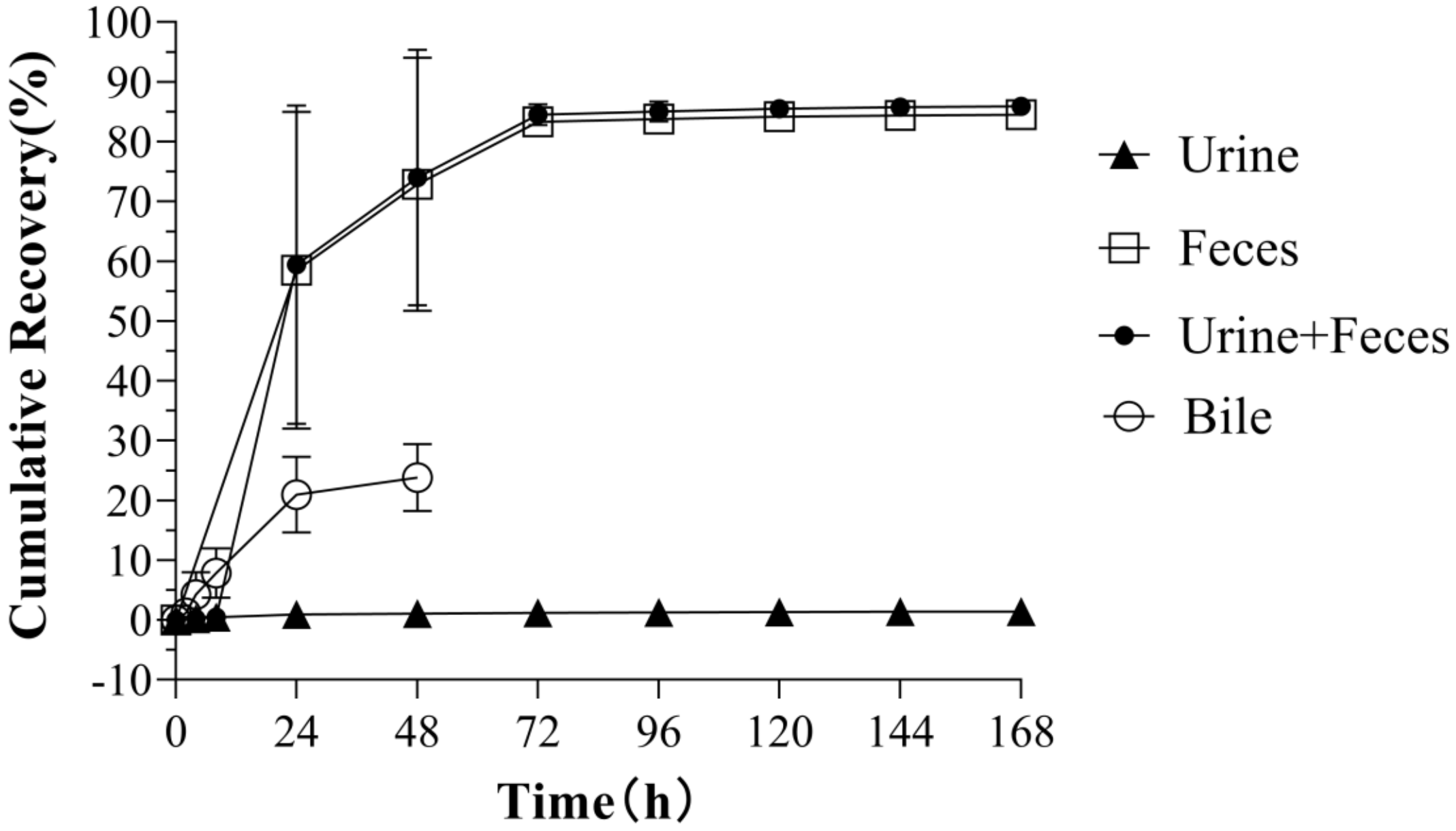

Supplementary Fig. 3 Cumulative radioactivity recovered from urine, feces and bile following a single oral dose of 6.96 MBq (188 μCi)/14.6 mg/kg [^{14}C]SH-1028 in rats (n=6). All data are presented as mean ± SD.

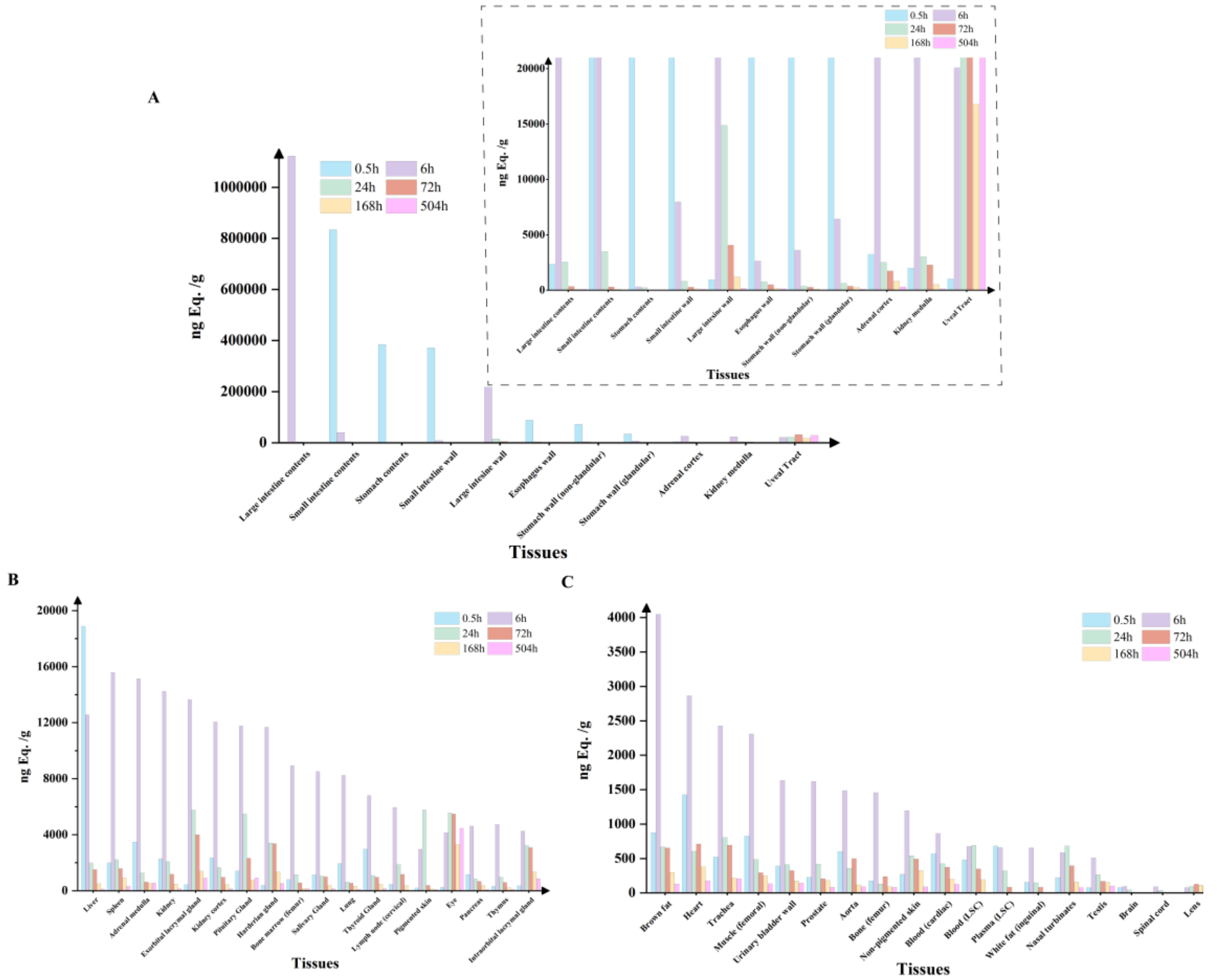

Supplementary Fig. 4 Radioactivity of selected tissues after a single oral dose of 3.7 MBq (100 μCi)/15 mg/kg [^{14}C]SH-1028 to male LE rats.

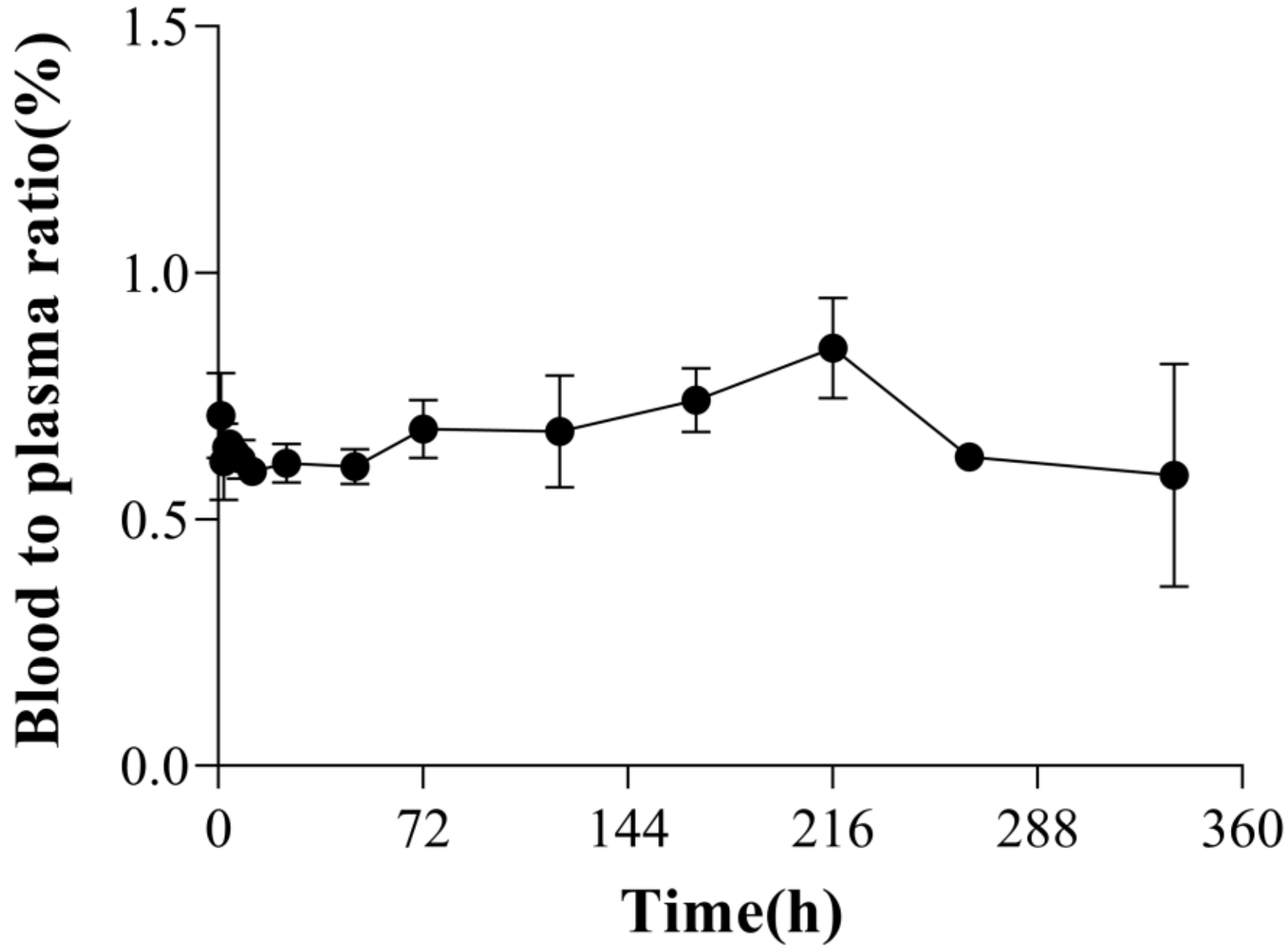

Supplementary Fig. 5 Blood to plasma ratio of radioactivity following a single oral dose of 200 mg containing 3.26 MBq (88 μCi) of [^{14}C]SH-1028 in healthy subjects (n=4).
